# Pneumococcal lineages associated with serotype replacement and antibiotic resistance in childhood invasive pneumococcal disease in the post-PCV13 era: an international whole-genome sequencing study

**DOI:** 10.1016/S1473-3099(19)30297-X

**Published:** 2019-07

**Authors:** Stephanie W Lo, Rebecca A Gladstone, Andries J van Tonder, John A Lees, Mignon du Plessis, Rachel Benisty, Noga Givon-Lavi, Paulina A Hawkins, Jennifer E Cornick, Brenda Kwambana-Adams, Pierra Y Law, Pak Leung Ho, Martin Antonio, Dean B Everett, Ron Dagan, Anne von Gottberg, Keith P Klugman, Lesley McGee, Robert F Breiman, Stephen D Bentley, The Global Pneumococcal Sequencing Consortium, Abdullah W Brooks, Alejandra Corso, Alexander Davydov, Alison Maguire, Andrew Pollard, Anmol Kiran, Anna Skoczynska, Benild Moiane, Bernard Beall, Betuel Sigauque, David Aanensen, Deborah Lehmann, Diego Faccone, Ebenezer Foster-Nyarko, Ebrima Bojang, Ekaterina Egorova, Elena Voropaeva, Eric Sampane-Donkor, Ewa Sadowy, Godfrey Bigogo, Helio Mucavele, Houria Belabbès, Idrissa Diawara, Jennifer Moïsi, Jennifer Verani, Jeremy Keenan, Jyothish N Nair Thulasee Bhai, Kedibone M Ndlangisa, Khalid Zerouali, K L Ravikumar, Leonid Titov, Linda De Gouveia, Maaike Alaerts, Margaret Ip, Maria Cristina de Cunto Brandileone, Md Hasanuzzaman, Metka Paragi, Michele Nurse-Lucas, Mushal Ali, Naima Elmdaghri, Nicholas Croucher, Nicole Wolter, Nurit Porat, Özgen Köseoglu Eser, Patrick E Akpaka, Paul Turner, Paula Gagetti, Peggy-Estelle Tientcheu, Philip E Carter, Rafal Mostowy, Rama Kandasamy, Rebecca Ford, Rebecca Henderson, Roly Malaker, Sadia Shakoor, Samanta Cristine Grassi Almeida, Samir K Saha, Sanjay Doiphode, Shabir A Madhi, Shamala Devi Sekaran, Somporn Srifuengfung, Stephen Obaro, Stuart C Clarke, Susan A Nzenze, Tamara Kastrin, Theresa J Ochoa, Veeraraghavan Balaji, Waleria Hryniewicz, Yulia Urban

**Affiliations:** a Parasites and Microbes, Wellcome Sanger Institute, Hinxton, UK; b Department of Microbiology, New York University School of Medicine, New York, NY, USA; c Centre for Respiratory Diseases and Meningitis, National Institute for Communicable Diseases, Johannesburg, South Africa; d The Faculty of Health Sciences, Ben-Gurion University of the Negev, Beer-Sheva, Israel; e Rollins School Public Health, Emory University, Atlanta, GA, USA; f Malawi-Liverpool-Wellcome-Trust, Blantyre, Malawi; g NIHR Global Health Research Unit on Mucosal Pathogens, Division of Infection and Immunity, University College London, London, UK; h WHO Collaborating Centre for New Vaccines Surveillance, Medical Research Council Unit The Gambia at The London School of Hygiene & Tropical Medicine, Fajara, The Gambia; i Department of Microbiology and Carol Yu Centre for Infection, The University of Hong Kong, Queen Mary Hospital, Hong Kong, China; j Centre for Inflammation Research, Queens Research Institute, University of Edinburgh, Edinburgh, UK; k Centers for Disease Control and Prevention, Atlanta, GA, USA; l Emory Global Health Institute, Emory University, Atlanta, GA, USA

## Abstract

**Background:**

Invasive pneumococcal disease remains an important health priority owing to increasing disease incidence caused by pneumococci expressing non-vaccine serotypes. We previously defined 621 Global Pneumococcal Sequence Clusters (GPSCs) by analysing 20 027 pneumococcal isolates collected worldwide and from previously published genomic data. In this study, we aimed to investigate the pneumococcal lineages behind the predominant serotypes, the mechanism of serotype replacement in disease, as well as the major pneumococcal lineages contributing to invasive pneumococcal disease in the post-vaccine era and their antibiotic resistant traits.

**Methods:**

We whole-genome sequenced 3233 invasive pneumococcal disease isolates from laboratory-based surveillance programmes in Hong Kong (n=78), Israel (n=701), Malawi (n=226), South Africa (n=1351), The Gambia (n=203), and the USA (n=674). The genomes represented pneumococci from before and after pneumococcal conjugate vaccine (PCV) introductions and were from children younger than 3 years. We identified predominant serotypes by prevalence and their major contributing lineages in each country, and assessed any serotype replacement by comparing the incidence rate between the pre-PCV and PCV periods for Israel, South Africa, and the USA. We defined the status of a lineage as vaccine-type GPSC (≥50% 13-valent PCV [PCV13] serotypes) or non-vaccine-type GPSC (>50% non-PCV13 serotypes) on the basis of its initial serotype composition detected in the earliest vaccine period to measure their individual contribution toward serotype replacement in each country. Major pneumococcal lineages in the PCV period were identified by pooled incidence rate using a random effects model.

**Findings:**

The five most prevalent serotypes in the PCV13 period varied between countries, with only serotypes 5, 12F, 15B/C, 19A, 33F, and 35B/D common to two or more countries. The five most prevalent serotypes in the PCV13 period varied between countries, with only serotypes 5, 12F, 15B/C, 19A, 33F, and 35B/D common to two or more countries. These serotypes were associated with more than one lineage, except for serotype 5 (GPSC8). Serotype replacement was mainly mediated by expansion of non-vaccine serotypes within vaccine-type GPSCs and, to a lesser extent, by increases in non-vaccine-type GPSCs. A globally spreading lineage, GPSC3, expressing invasive serotypes 8 in South Africa and 33F in the USA and Israel, was the most common lineage causing non-vaccine serotype invasive pneumococcal disease in the PCV13 period. We observed that same prevalent non-vaccine serotypes could be associated with distinctive lineages in different countries, which exhibited dissimilar antibiotic resistance profiles. In non-vaccine serotype isolates, we detected significant increases in the prevalence of resistance to penicillin (52 [21%] of 249 *vs* 169 [29%] of 575, p=0·0016) and erythromycin (three [1%] of 249 *vs* 65 [11%] of 575, p=0·0031) in the PCV13 period compared with the pre-PCV period.

**Interpretation:**

Globally spreading lineages expressing invasive serotypes have an important role in serotype replacement, and emerging non-vaccine serotypes associated with different pneumococcal lineages in different countries might be explained by local antibiotic-selective pressures. Continued genomic surveillance of the dynamics of the pneumococcal population with increased geographical representation in the post-vaccine period will generate further knowledge for optimising future vaccine design.

**Funding:**

Bill & Melinda Gates Foundation, Wellcome Sanger Institute, and the US Centers for Disease Control.

## Introduction

Pneumococcal conjugate vaccines (PCVs), thus far targeting up to 13 pneumococcal serotypes that account for most of the disease in infants, have substantially reduced the global burden of invasive pneumococcal disease in children.^1^, ^2^, ^3^, ^4^, ^5^ Despite the success of PCVs, invasive pneumococcal disease remains an important health priority owing to an increase in the incidence of disease caused by non-vaccine serotypes, an event known as serotype replacement. Serotype replacement has been reported for several countries, but not the USA, after the introduction of the 13-valent PCV (PCV13), and the magnitude of increases in non-vaccine serotypes varies by country.^1^, ^2^, ^3^, ^6^, ^7^, ^8^ Although studies^3^, ^9^, ^10^, ^11^, ^12^ characterising serotype replacement in cases of invasive pneumococcal disease have primarily focused on changes in serotypes, mechanisms by which the pneumococcal population evolves and adapts to the selective pressure of PCVs, and the relative contribution of each mechanism to the increase in non-vaccine serotypes, are not completely understood.

**Research in context**

**Evidence before this study**

We searched PubMed, using the terms “pneumococcal conjugate vaccine” AND “genome” OR “genotype” OR “clone”, for papers published in English between Jan 1, 2000, and Aug 21, 2018. We searched for population-based studies of invasive pneumococcal disease that reported changes in serotype and genotype (or clone) before and after pneumococcal conjugate vaccine (PCV) introduction. After reviewing 288 articles, 21 met inclusion criteria. The effects of 7-valent PCV (PCV7) were measured in 11 studies, 10-valent PCV (PCV10) in three, and 13-valent PCV (PCV13) in seven. These studies were done at the national or regional level, and the majority delineated genotypes using multilocus sequence typing or pulsed-field gel electrophoresis. Only one study from the USA, which reported the post-PCV7 changes using 349 isolates, delineated pneumococcal genotypes using whole-genome sequences. No published reports were available investigating the changes in both serotype and clone before and after PCV10 or PCV13 introduction from an international perspective. Our accompanying paper reported an international definition of pneumococcal lineages or Global Pneumococcal Sequence Clusters (GPSCs) using 20 027 pneumococcal genomes. This is the first study to make full use of whole-genome sequences to delineate pneumococcal lineages with a standardised clustering nomenclature. The GPSC reference database can be updated over time to accommodate novel GPSCs identified in the future. This genomic typing scheme is key to contextualise disease, antibiotic resistance, and vaccine effect at a local and international level.

**Added value of this study**

We used the international definition of Global Pneumococcal Sequence Clusters to assess the effects of PCVs on the population structure of invasive pneumococcal disease across several countries. This standardised and high-resolution typing scheme allows for the identification of key international lineages driving serotype replacement in disease and changes in the prevalence of antibiotic resistance in the post-PCV13 era.

**Implications of all the available evidence**

Use of whole-genome sequencing and epidemiological data in pneumococci surveillance programmes provides enhanced understanding of the changes in pneumococcal serotypes, genotypes, and antimicrobial resistance nationally and internationally after the introduction of PCV13. Provision of a user-friendly web tool that enables analysis of new genomic data with rapid GPSC assignment, serotyping, and antimicorbial resistance prediction is key for ongoing genomic surveillance. We await confirmation of the pneumococcal lineages we found to be associated with serotype replacement from surveillance programmes that extend beyond 4 years post-PCV13 with greater geographical representation. The knowledge generated should drive effective next-generation vaccine design.

Two mechanisms of serotype replacement have been suggested. The first is expansion of non-vaccine-type lineages to partly fill the niche vacated by vaccine-type lineages.^13^ The second is within-lineage expansion of non-vaccine serotypes, which can be explained as a concomitant increase in non-vaccine serotypes and decrease in vaccine serotypes within a lineage after PCV introduction, usually through expansion of existing non-vaccine-type components rather than contemporaneous capsular switching.^14^, ^15^ Multiple serotypes (vaccine and non-vaccine) expressed by a single pneumococcal lineage were not unusual^14^, ^16^ because pneumococci is able to replace capsular biosynthesis genes through acquisition of foreign DNA by natural genetic transformation.^17^

The Global Pneumococcal Sequencing (GPS) project was set up to guide the design of preventive measures for pneumococcal diseases beyond PCV13 by defining pneumococcal lineages across a global pneumococcal population and attempting to capture how lineages evade vaccines. As part of the GPS project, a high-resolution population structure of 621 Global Pneumococcal Sequence Clusters (GPSCs) was defined by analysing 20 027 pneumococci genomes.^18^ In this study, we aimed to identify the predominant serotypes causing invasive pneumococcal disease among children younger than 3 years and their associated lineages after the introduction of PCV13 in six countries, investigate the mechanisms of serotype replacement in disease, and identify international lineages contributing to post-PCV13 invasive pneumococcal disease and report their antibiotic-resistant traits.

## Methods

### Study design

From the GPS database (last accessed June 1, 2017), we extracted genomic data from invasive isolates causing disease in children younger than 3 years in six countries (Hong Kong, Israel, Malawi, South Africa, The Gambia, and the USA). For each country, the collection represented pneumococci from before and after the introduction of 7-valent PCV (PCV7) and PCV13 into the universal immunisation programmes.

An invasive pneumococcal disease case was defined as pneumococci cultured from a normally sterile site. The overall GPS database comprises 13 454 genomes, including 8605 disease and 4849 carriage isolates, representing 30 countries from Africa (n=7928), Asia (n=2381), North America (n=1684), South America (n=1027), and Europe (n=434). Collections from countries other than the six included in this study either did not have 50 or more isolates from before and after the introduction of PCVs, or were biased by sampling a particular serotype, and were thus not included in this analysis (appendix 1). Pneumococcal isolates collected from children younger than 3 years were selected because this age group represents the primary target for vaccination; any serotype and clonal changes in response to vaccine-induced selective pressure are expected to be most quickly reflected in this population. The vaccination schedule, catch-up scheme, and PCV uptake for each of the six countries are summarised in table 1.

**Table 1:** Characteristics of country-specific datasets containing pneumococcus invasive isolates from children younger than 3 years

		**China**	**Israel**	**Malawi**	**South Africa**	**The Gambia**	**USA**
Regions		Hong Kong	Nationwide	Blantyre	Nationwide	Nationwide	10 of 50 states*
World Bank income group		High income	High income	Low income	Upper-middle income	Low income	High income
Year of PCV7 introduction (vaccine schedule†)		2009 (3 + 1, with catch-up)	2009 (2 + 1, with catch-up)	No PCV7 introduction	2009 (2 + 1, without catch-up)	2009 (3 + 0, without catch-up)	2000 (3 + 1, with catch-up)
Year of PCV10 or PCV13 introduction (vaccine schedule)		PCV10 in 2010 (3 + 1, with catch-up); PCV13 in 2011 (3 + 1, with catch-up)	PCV13 in 2010 (2 + 1, without catch-up)	PCV13 in 2011 (3 + 0, without catch-up)	PCV13 in 2011 (2 + 1, with limited catch-up for children <30 months old)	PCV13 in 2011 (3 + 0, without catch-up)	PCV13 in 2010 (3 + 1, with catch-up)
PCV13 uptake during the study period‡		95%	92–94%	87–88%	69–72%	96–97%	92%
Number of isolates (collection period)							
	Pre-PCV	45 (1995–2001)	357 (2005–09)	213 (1997–2011)	763 (2005–09)	62 (1996–2009)	137 (1998–99)
	PCV7	13 (2010–11)	67 (2010)	None	297 (2010–11)	55 (2010–11)	410 (2001–09)
	PCV13	20 (2012–15)	277 (2011–14)	13 (2014–15)	291 (2013–14)	86 (2013–14)	127 (2012)
	Total	78	701	226	1351	203	674

PCV=pneumococcal conjugate vaccine. PCV7=7-valent PCV. PCV10=10-valent PCV. PCV13=13-valent PCV.

* California, Colorado, Connecticut, Georgia, Maryland, Minnesota, New Mexico, New York, Oregon, and Tennessee.^19^

† The 3 + 1 immunisation schedule consisted of a standard three-dose primary series at 2, 4, and 6 months of age and a booster dose at 12–15 months; the 2 + 1 schedule consisted of two-dose primary series at 2 and 4 months of age and a booster dose at 12 months in Israel and at 6 and 14 weeks of age and a booster dose at 9 months of age in South Africa; the 3 + 0 immunisation schedule consisted of three-dose primary series at 6, 10, and 14 weeks of age in Malawi and at 2, 3, and 4 months of age in The Gambia. Catch-up immunistaion was given to children whose vaccination had been delayed.

‡ PCV13 uptake in the study period was based on WHO statistics.^20^

### Data processing

The pnuemococcal isolates in the GPS database were whole-genome sequenced on an Illumina HiSeq Sequencer. We included the pneumococcal genomes from the GPS database on the basis of the aforementioned criteria and analysed them as previously described.^18^ In brief, pneumococcal isolates were clustered into GPSCs on the basis of their shared sequence and gene content using a fast and flexible bacterial genomic software, Population Partitioning Using Nucleotide K-mers (PopPUNK).^21^ Serotypes were typed in silico,^22^, ^23^ and antibiotic susceptibility for penicillin (encoded by the genes *pbp*1A, *pbp*2B, and *pbp2A*), chloramphenicol (*cat*), erythromycin (*ermB, ermTR1*, and *mefA*), cotrimoxazole (*folA* and *folP)*, and tetracycline (*tet*[M], *tet*[O], and *tet*[S/M]) was predicted using the sequencing reads and the published tools developed by the USA Centers for Disease Control.^24^, ^25^, ^26^ The genome sequences were deposited in the European Nucleotide Archive.

We compiled metadata, including age, year of collection, and sample source, from the GPS database. On the basis of the year of PCV introduction, we grouped isolates into three vaccine periods: those from the pre-PCV period (years when no conjugate vaccine was used and the year of first PCV introduction), those from the PCV7 period (from the second year of PCV7 introduction to the first year of PCV13 introduction), and those from the PCV13 period (from the second year of PCV13 introduction to the end of the collection year). Serotypes were grouped into two categories: vaccine serotypes (1, 3, 4, 5, 6A, 6B, 7F, 9V, 14, 18C, 19A, 19F, and 23F) and non-vaccine serotypes (serotypes not included in PCV13 and non-typeable). We defined the status of a lineage as vaccine serotype GPSC (≥50% PCV13 serotypes) or non-vaccine serotype GPSC (>50% non-PCV13 serotypes) on the basis of its initial serotype composition detected in the earliest vaccine period. The GPSC assignments, in-silico serotype, genotypic antimicrobial resistance, European Nucleotide Archive accession numbers, and metadata are collated in appendix 2.

### Statistical analysis

Data on the annual incidence of invasive pneumococcal disease before and after the introduction of PCV7 or PCV13 was available for South Africa, Israel, and the USA. For each of these three countries, we used the Poisson regression to calculate the incidence rate ratios (IRRs) of total invasive pneumococcal disease, non-vaccine-type invasive pneumococcal disease, or individual GPSC using the estimated incidence rate of invasive pneumococcal disease per year for each of the two vaccine periods.^27^ If overdispersion was detected, we reported the robust standard error for minor violation of the Poisson regression assumption measured by goodness-of-fit (p value ranged between 0·01 and 0·05) or used the negative bionomial regression (Poisson goodness-of-fit p value <0·01). If neither model fit, we calculated IRRs using the average annual incidence for the two periods. To avoid IRRs being calculated as zero or infinity, we added a constant number of one to all the estimated invasive pneumococcal disease cases for both periods if a GPSC was not observed in one of the two PCV periods. Serotype replacement was detected if the IRR and lower bound for 95% CI were above one.^28^ By use of the random effects model in the R meta package,^29^ a pooled incidence rate of non-vaccine-type invasive pneumococcal disease for each GPSC was calculated for Israel, South Africa, and USA.

For Hong Kong, Malawi, and The Gambia, increase of a particular non-vaccine serotype in the overall population or within a GPSC was calculated as a proportion of all non-vaccine serotypes and compared between vaccine periods using Fisher's exact test. For these countries, incidence rates could not be calculated owing to unavailable information about the actual number of invasive pneumococcal disease cases per year and estimates of the child population in the study period.

A generalised linear binomial regression was used to detect changes in antimicrobial non-susceptibility to penicillin, chloramphenicol, erythromycin, cotrimoxazole, and tetracycline between the pre-PCV and PCV13 periods, where country was included as a dependent variable to account for variations in resistance prevalence between countries. Odds ratios indicating the invasive disease potential of serotypes were calculated previously,^18^ whereby a country-matched and year-matched collection of disease (n=1081) and carriage (n=1434) isolates from children younger than 7 years was used to calculate a pooled odds ratio with a meta-analysis approach.^18^, ^30^, ^31^ The invasive disease potential of serotypes measured in the child population younger than 7 years was shown to be conserved in the child population younger than 3 years.^32^ Two-sided p values of less than 0·05 were considered significant. Multiple testing correction was done using the Benjamini-Hochberg false discovery rate of 5% when number of tests is greater than ten. Statistical tests were done in R version 3.5.2. R scripts used for analyses are available in a public depository. Further details of the methods are included in appendix 1.

### Role of the funding source

The funders of the study had no role in the study design, data collection, data analysis, data interpretation, or writing of the report. The corresponding author had full access to all the data in the study and had final responsibility for the decision to submit for publication.

## Results

Of the 8605 disease isolates in the GPS database, 4416 were from children younger than 3 years from 27 countries (appendix 1). Of these, we included whole-genome sequencing data from 3233 isolates collected from Hong Kong (n=78), Israel (n=701), Malawi (n=226), South Africa (n=1351), The Gambia (n=203), and the USA (n=674; table 1). For the PCV13 period, sequences were available for 1 year in the USA, 2 years in Malawi, South Africa, and The Gambia, and 4 years in Israel and Hong Kong (table 1).

**Figure 1:**
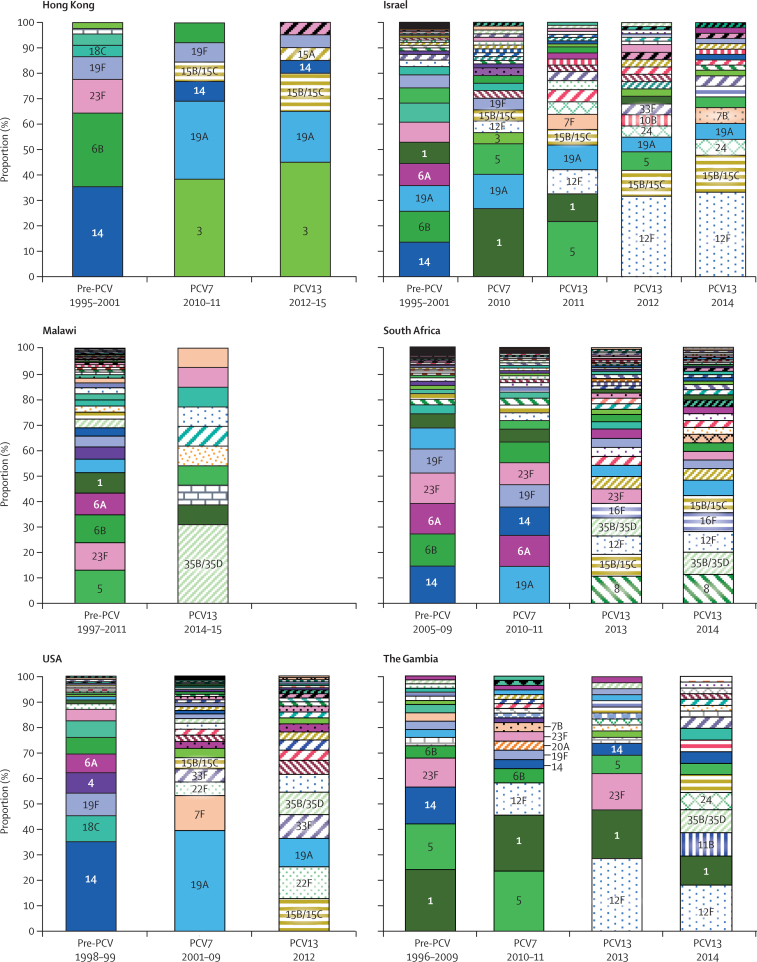
Serotype distribution among invasive pneumococcal isolates from children younger than 3 years before and after the introduction of PCV13 Vaccine serotypes are represented by solid fill and non-vaccine-serotypes by coloured hatched patterns. The five most prevalent serotypes for each period are labelled. Serotypes with fewer than two isolates are not labelled. PCV=pneumococcal conjugate vaccine. PCV7=7-valent PCV. PCV13=13-valent PCV.

**Figure 2:**
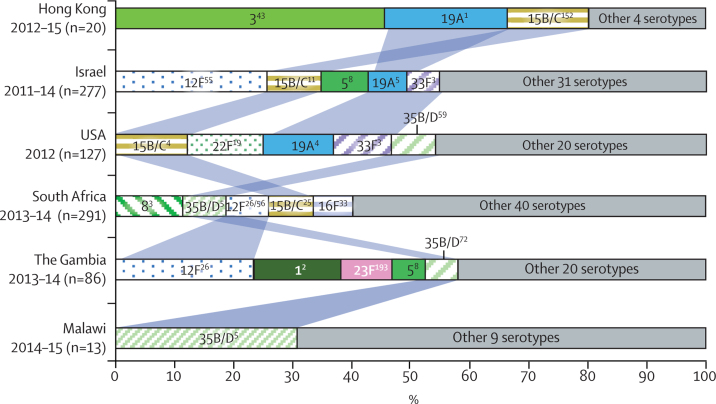
Comparison of the five most prevalent pneumococcal serotypes in Global Pneumococcal Sequencing key sites in the PCV13 period The five most prevalent serotypes are indicated in boxes in proportion to their prevalence. The predominant Global Pneumococcal Sequence Clusters (GPSCs) associated with these serotypes are written in superscript, and identical GPSCs are written in same colour. Blue bands indicate the serotypes shared between countries. Serotypes with fewer than two isolates are not listed. PCV13=13-valent pneumococcal conjugate vaccine.

Before the introduction of PCV, five PCV7 serotypes (6B, 14, 18C, 19F, and 23F) and three PCV13 serotypes (1, 5, and 6A) were prevalent in two or more countries. Apart from Malawi, we observed changes in major serotypes in the PCV7 period, in which PCV7 serotypes (14, 19F, 23F), PCV13 unique serotypes (1, 3, 5, 19A), and non-vaccine serotypes (15B/C and 12F) were among the five most common to at least two countries (figure 1). Following the introduction of PCV13, invasive pneumococcal disease caused by vaccine-type pneumococci significantly decreased in incidence in Israel, South Africa, and the USA and in prevalence for Hong Kong, Malawi, and The Gambia (appendix 1). Non-vaccine-type pneumococci accounted for the majority of invasive pneumococcal disease cases in all countries in the PCV13 period, except for Hong Kong where PCV13 serotypes 3 and 19A still accounted for 13 (65%) of 20 cases (Figure 1, Figure 2; appendix 1). The five most prevalent serotypes (vaccine-type and non-vaccine type) in the PCV13 period varied between countries, but serotypes 5, 12F, 15B/C, 19A, 33F, and 35B/D were common to two or more countries (figure 2; appendix 1).

The major lineages associated with these shared serotypes also differed between countries, except for lineages GPSC3, GPSC5, GPSC8, and GPSC26 (figure 2). In the PCV13 period, GPSC3 was present in three of six countries, accounting for all invasive pneumococcal disease cases caused by serotype 8 pneumococci (the most common serotype) in South Africa and all disease cases caused by serotype 33F (the fourth most common) in the USA and Israel (the fifth most common). GPSC5 was also found in three of six countries, predominantly expressing 35B/D in South Africa and Malawi but serotype 19A in Israel. It contributed to 19 (83%) of the 23 serotype 35B/D invasive pneumococcal disease isolates in South Africa (ranked second) in the PCV13 period, all four 35B/D isolates in Malawi (ranked first), and nine (53%) of 17 serotype 19A isolates (ranked fourth) in Israel. GPSC8, which exclusively expressed serotype 5, represented all serotype 5 isolates in The Gambia (n=5) and Israel (n=26) in the PCV13 period. GPSC26 was mainly composed of serotype 12F (34 [97%] of 35) and accounted for all serotype 12F isolates (n=20, ranked first) in The Gambia and ten (45%) of 22 serotype 12F isolates (ranked second) in South Africa in the PCV13 period. A few isolates from GPSC26 were also observed in Malawi (n=1) and Israel (n=3) in the PCV13 period.

Serotype replacement was detected in Israel (IRR 2·1, 95% CI 1·6–2·9, p<0·0001), but not in South Africa (1·2, 0·8–1·7, p=0·35) or the USA (1·1, 0·5–2·4, p=0·84). The increases in non-vaccine-type invasive pneumococcal disease did not offset the overall invasive pneumococcal disease reductions in these three countries (IRR 0·42, 95% CI 0·34–0·43 for Israel; 0·27, 0·23–0·30 for South Africa; 0·08, 0·07–0·10 for the USA; p<0·0001 for all comparisons; appendix 1). We observed significant increases in incidence of non-vaccine-type GPSCs in Israel and increases in incidence of non-vaccine-type components within vaccine-type GPSCs in Israel and South Africa (table 2).

**Table 2:** Individual contribution of the two mechanisms toward serotype replacement in IPD

	**Israel**	**South Africa**	**USA**
Mechanism 1*			
Number of non-VT GPSCs	36	66	24
Non-VT IPD cases caused by non-VT GPSCs in the pre-PCV period	49/56 (88%)	111/122 (91%)	7/11 (64%)
Non-VT IPD cases caused by non-VT GPSCs in the PCV13 period†	153/195 (78%)	168/209 (80%)	63/106 (59%)
Incidence of non-VT IPD caused by non-VT GPSCs in the pre-PCV period, per 100 000 children	8·2	4·5	6·8
Incidence of non-VT IPD caused by non-VT GPSCs in the PCV13 period, per 100 000 children	15·8	4·7	6·0
IRR (95% CI, p value)‡	1·9 (1·4–2·6, p<0·0001)	1·1 (0·7–1·6, p=0·78)	0·9 (0·7–1·2, p=0·40)
**Mechanism 2**§			
Number of VT GPSCs	47	53	29
Non-VT IPD cases caused by VT GPSCs in the pre-PCV period	7/56 (12%)	11/122 (9%)	4/11 (36%)
Non-VT IPD cases caused by VT GPSCs in the PCV13 period†	42/195 (22%)	41/209 (20%)	43/106 (41%)
Incidence of non-VT IPD caused by VT GPSCs in the pre-PCV period, per 100 000 children	1·3	0·4	2·6
Incidence of non-VT IPD caused by VT GPSCs in the PCV13 period, per 100 000 children	4·2	1·1	4·1
IRR (95% CI, p value)‡	3·4 (1·8–6·3; p<0·0001)	2·5 (1·6–3·9; p<0·0001)	1·6 (1·0–2·4; p=0·12)

VT=vaccine type. GPSC=Global Pneumococcal Sequence Cluster. IPD=invasive pneumococcal disease. PCV=pneumococcal conjugate vaccine. IRR=incidence rate ratio.

* Increase in disease caused by non-VT GPSCs (ie, those expressing >50% non-vaccine serotypes in the pre-PCV13 period).

† The PCV13 period represented 4 years (2011–14) in Israel, 2 years (2013–14) in South Africa, and 1 year (2012) in the USA.

‡ IRRs were calculated with the estimated incidence of non-VT IPD caused by VT or non-VT GPSCs per year for the pre-PCV and PCV13 periods.

§ Increase in non-VT component within VT GPSCs (ie, those expressing ≥50% vaccine serotype in the pre-PCV period).

Pooling GPSCs by their classification as either non-vaccine type or vaccine type, we observed that more non-vaccine-type GPSCs than vaccine-type GPSCs contributed to cases of non-vaccine-type invasive pneumococcal disease in Israel, South Africa, and the USA in the PCV13 period, although the magnitude of increase in disease caused by non-vaccine serotypes (measured by IRR) was larger for vaccine-type than for non-vaccine-type GPSCs (table 2). The contribution of either mechanism varied between countries (table 2; appendix 1).

In the PCV13 period, eight non-vaccine-type GPSCs increased in incidence (GPSC3, 59, 55, 168, 25, 139, and 132, descending order of pooled incidence rate) or in prevalence (GPSC5). Significant increases in incidence of non-vaccine serotypes occurred within three vaccine-type GPSCs (GPSC11, 5, and 6; table 3). Among them, the GSPC5 lineage was observed to be significantly expanding in more than one country in the PCV13 period; the expansions of other GPSCs were country-specific (table 3). We also observed that lineages expressing the same serotypes could change differently after the introduction of PCV13 within a country. For example, of the two lineages in Israel expressing serotype 12F in the PCV13 period (GPSC26 and GPSC55), only GPSC55 significantly increased in that period (appendix 1).

**Table 3:** GPSCs causing non-VT IPD in the PCV13 era

	Pooled incidence rate of non-VT IPD (95% CI)*	**Number of non-VT isolates**†	**Mechanism 1**‡	**Mechanism 2**§	**Penicillin resistance, n (%)**	**Not susceptible to two or more classes of antibiotics, n (%)**
**Ten most prevalent GPSCs causing non-VT IPD in the PCV13 era**						
GPSC3	2·70 (1·34–5·42)	71	8 (South Africa)¶	..	4 (6%)	15 (21%)
GPSC19	0·89 (0·28–2·82)	21	..	..	0	0
GPSC11	0·86 (0·06–12·61)	32	..	19A→15B/C (Israel)	1 (3%)	1 (3%)
GPSC5	0·66 (0·27–1·62)	28	35B/D (Malawi)	23F→35B/D (South Africa)¶	26 (93%)	24 (86%)
GPSC38	0·65 (0·11–3·94)	17	..	..	0	0
GPSC26	0·58 (0·21–1·58)	35	..	..	0	35 (100%)
GPSC36	0·49 (0·20–1·22)	12	..	..	0	0
GPSC48	0·48 (0·12–1·97)	11	..	..	11 (100%)	7 (64%)
GPSC9	0·47 (0·05–4·00)	13	..	..	13 (100%)	13 (100%)
GPSC7	0·42 (0·07–2·64)	10	..	..	0	0
**Other important GPSCs in the PCV13 era**						
GPSC59	0·32 (0·06–1·75)	13	35B/D (USA)	..	13 (100%)	10 (77%)
GPSC55	0·29 (0·00–80·15)	65	12F (Israel)	..	65 (100%)	0
GPSC168	0·22 (0·09–0·50)	5	15A (South Africa)	..	5 (100%)	5 (100%)
GPSC25	0·21 (0·03–1·40)	12	15B/C (South Africa)	..	0	0
GPSC6	0·16 (0·03–0·82)	8	..	9V→15B/C (USA)¶	2 (25%)	2 (25%)
GPSC139	0·16 (0·00–8·87)	9	10B (Israel)	..	0	0
GPSC132	0·12 (0·01–1·04)	5	15B/C (USA)¶	..	5 (100%)	5 (100%)

Serotypes with a significantly higher invasive disease potential (odds ratio for invasiveness >1, p value <0·05)^18^ were 8, 19A, and 12F. Serotypes with a significantly lower invasive disease potential (odds ratio for invasiveness <1, p value <0·05) were 15A, 15B/C, 23B, 21, and 35B. VT=vaccine serotype. IPD=invasive pneumococcal disease. PCV=pneumococcal conjugate vaccine. GPSC=Global Pneumococcal Sequence Cluster.

* Calculated for Israel, South Africa, and the USA, where annual incidence of IPD was available; data are per 100 000 children.

† Number of isolates from all six countries in the PCV13 period.

‡ Increase in disease caused by non-VT GPSCs (ie, those expressing >50% non-vaccine serotypes in the pre-PCV13 period) in incidence for Israel, South Africa, and the USA, and in prevalence for Hong Kong, Malawi, and The Gambia.

§ Increase in non-VT component within VT-GPSCs (ie, those expressing ≥50% vaccine serotype in the pre-PCV period); data are presented as predominant serotype in the pre-PCV period → predominant serotype in the PCV13 period (country).

¶ Significant increase was observed only between the PCV7 and PCV13 periods.

Pooled incidence rates of the ten most prevalent international lineages causing non-vaccine-type invasive pneumococcal disease in the PCV13 period are listed in table 3. GPSC3, which mainly expressed two invasive serotypes (8 and 33F),^18^ was the predominant lineage causing non-vaccine-type invasive pneumococcal disease in the PCV13 period. GPSC26 (ranked sixth) was another lineage expressing invasive serotypes: of the 63 isolates from all vaccine periods, 57 (90%) were serotype 12F and six (10%) were serotype 46. In The Gambia, GPSC26 accounted for all serotype 12F isolates and became the leading cause of invasive pneumococcal disease in the PCV13 period. The other eight lineages either expressed non-vaccine serotypes that were significantly associated with carriage (GPSC11, 48, and 9) or non-vaccine serotypes that were not significantly associated with disease or carriage (table 3).^18^

The introduction of PCV13 was associated with overall reductions in the prevalence of resistance to all classes of antibiotics, except to chloramphenicol, which was maintained at around 5% throughout the vaccine periods (table 4). Among the non-vaccine-type isolates, we detected significant increases in penicillin and erythromycin resistance and a significant decrease in cotrimoxazole resistance between the pre-PCV and PCV13 periods (table 4). Of the ten most common GPSCs, three (GPSC5, 48, and 9) had a prevalence of penicillin resistance ≥90% and four (GPSC5, 26, 48, and 9) had 64–100% of isolates non-susceptible to at least two classes of antibiotics (table 3). Isolates belonging to three non-vaccine-type GPSCs (GPSC55, 59, 168, and 132) that significantly increased in incidence between the pre-PCV and PCV13 periods were 100% resistant to penicillin. GPSC59 also showed *mefA*-mediated erythromycin resistance, GPSC132 was additionally resistant to *mefA*-mediated erythromycin and tetracycline resistance, and GPSC168 was additionally resistant to cotrimoxazole (appendix 1). Of note, GPSC26 exhibited non-susceptibility to chloramphenicol (31 [89%] of 35), tetracycline (32 [91%] of 35), and cotrimoxazole (35 [100%] of 35; appendix 1), although remained susceptible to penicillin (table 3).

**Table 4:** Changes in prevalence of antibiotic non-susceptibility between pre-PCV and PCV13 periods

		**Number of isolates (%)**		Adjusted p value by linear regression*
		Pre-PCV period	PCV13 period	
Overall (n)		1577	814	..
	Penicillin	774 (49%)	277 (34%)	<0·0001
	Chloramphenicol	78 (5%)	39 (5%)	0·93
	Erythromycin	377 (24%)	122 (15%)	<0·0001
	Cotrimoxazole	1118 (71%)	399 (49%)	<0·0001
	Tetracycline	446 (28%)	148 (18%)	<0·0001
	Multidrug resistance†	410 (26%)	125 (15%)	<0·0001
Non-VT isolates only (n)		249	575	..
	Penicillin	52 (21%)	169 (29%)	0·0016
	Chloramphenicol	14 (6%)	31 (5%)	0·33
	Erythromycin	3 (1%)	65 (11%)	0·0031
	Cotrimoxazole	120 (48%)	224 (39%)	0·021
	Tetracycline	36 (14%)	80 (14%)	0·83
	Multidrug resistance†	21 (8%)	59 (10%)	0·79

PCV=pneumococcal conjugate vaccine. PCV13=13-valent pneumococcal conjugate vaccine. VT=vaccine serotype.

* A generalised linear regression to detect the significant correlation between PCV13 introduction and changes in antibiotic prevalence after accounting for the variations between countries.

† Multidrug resistance was defined as isolates non-susceptible to three or more classes of antibiotics detected in this study.

In the PCV13 period, we observed differences in prevalence of antibiotic non-susceptibility between countries within a lineage (appendix 1). Compared with other countries, GPSC3 and GPSC5 isolates from the USA had a higher prevalence of resistance to erythromycin, mostly (13 [87%] of 15) mediated by *mefA*, whereas isolates from Israel belonging to GPSC3 had a higher prevalence of resistance to penicillin (appendix 1). For GPSC3, the prevalence of erythromycin resistance mediated by *mefA* in the USA was zero in the pre-PCV period, but increased to 46% (12 of 26) in the PCV7 period and to 67% (12 of 18) in the PCV13 period (pre-PCV *vs* PCV13 period, p=0·0287). By contrast, changes in cotrimoxazole resistance in that lineage were not significant (appendix 1). In Israel, prevalence of penicillin resistance in GPSC3 did not significantly change between pre-PCV (five [45%] of 11) and PCV7 (one [50%] of two, p=1·000) or PCV13 (four [29%] of 14, p=0·4341) periods.

## Discussion

Our analyses showed that globally spreading lineages expressing invasive serotypes had an important role in serotype replacement. The high-resolution and standardised population structure allowed us to understand the lineages that drove the increases in non-vaccine-type invasive pneumococcal disease beyond serotypes at local and international levels.

We found that the same serotype could be associated with different major lineages in different countries (eg, serotype 12F was associated with GPSC55 in Israel but GPSC26 in The Gambia and South Africa). Such variations might be explained by differences in the initial bacterial population, host characteristics, or antibiotic selective pressures between countries. For example, although GPSC26 persisted in Israel without significant changes in incidence between pre-PCV and PCV13 periods, GPSC55, with 100% resistance to penicillin, significantly increased in Israel in that time, which might be due to the common use of β-lactam antibiotics for treating pneumococcal disease in the country.^33^ By contrast, GPSC26, with cotrimoxazole resistance but susceptibility to penicillin, was the predominant lineage, accounting for most of the serotype 12F isolates, in The Gambia and South Africa, where cotrimoxazole is widely used to prevent bacterial infection in people with HIV.^34^, ^35^ In South Africa, we observed that a serotype 15B/C lineage (GPSC25) replaced another serotype 15B/C lineage (GPSC48) during the introduction of PCVs; an event known as clonal replacement. Clonal replacement was also reported among serotype 19A pneumococci in the USA following the introduction of PCV7, in which sequence type (ST) 320 outcompeted ST199.^36^ Further investigation into the genomic differences between the two STs might help explain these observations.

We found that major lineages could be associated with different serotypes in different countries, such as lineages GPSC3 (eg, serotype 8 in South Africa and 33F in the USA), GPSC5, and GPSC11. Before the introduction of PCVs, high serotype diversity was already observed in these three lineages, with the highest in GPSC11 (Simpson's diversity index 0·84, ranked the first of 621 lineages in our previous study), followed by GPSC5 (0·75, ranked second) and GPSC3 (0·72, ranked fifth).^18^ GPSC3 and GPSC5 were reported to be the cause of invasive pneumococcal disease internationally before the introduction of PCVs and classified by the Pneumococcal Molecular Epidemiology Network as PMEN33 and PMEN26, respectively.^37^ Such established international lineages are important to consider when attempting to identify a global solution for the control of pneumococcal disease.

Non-vaccine-type GPSCs, rather than the vaccine-type GPSCs with a non-vaccine-type component, accounted for the largest proportion (>59%) of invasive pneumococcal disease caused by non-vaccine serotypes after the introduction of PCV13. However, we observed that only eight non-vaccine-type lineages increased significantly in this dataset following the introduction of PCV13. This finding suggests that non-vaccine-type lineages that persisted in the population without increasing in prevalence also had an important part in causing disease when invasive pneumococcal disease caused by vaccine serotypes decreased. For example, a lineage expressing serotype 22F, GPSC19, ranked second in contribution to non-vaccine-type invasive pneumococcal disease in the PCV13 period but was not detected to have increased in either Israel or the USA. Of note, such non-vaccine-type lineages (GPSC19, GPSC38, GPSC48, GPSC9, GPSC7, and GPSC36; appendix 1) were found in several countries and expressed serotypes that were observed to be major non-vaccine serotypes causing invasive pneumococcal disease in multiple countries after PCV13 introduction.^3^, ^6^, ^38^, ^39^ The application of GPSCs to isolates from additional countries could allow better understanding of these non-vaccine serotypes importance to disease replacement.

Among the ten most prevalent lineages causing non-vaccine-type invasive pneumococcal disease in the PCV13 period, two (GPSC3 and GPSC26) expressed invasive serotypes 8, 12F, and 33F. Increases in serotype 12F resulting in disease replacement were reported in England and Wales,^6^ France,^9^, ^40^ and Germany,^39^ although the genotypes of those isolates were not reported. Globally, serotype 12F isolates mainly belong to clonal complex (CC) 989 (GPSC26),^41^, ^42^ but also to ST218 (GPSC32) in North America^43^, ^44^ and CC3524 (GPSC55), a regional lineage, in Israel. Increase in serotype 8 was also observed among children less than 5 years of age in England and Wales in 2016-17.^6^ A 10-year invasive pneumococcal disease study^45^ done in Oxfordshire in 1996–2005 showed that all serotype 8 disease isolates were ST53 (GPSC3); therefore, GPSC3 might also be involved in disease replacement in England and Wales. In the post-PCV13 era, invasive serotype 33F pneumococci were reported to be one of the major non-vaccine types causing childhood invasive pneumococcal disease in North America;^46^ they were predominantly CC100 (GPSC3) in Canada and the USA and associated with erythromycin resistance.^47^, ^48^ Invasive disease potential for serotype 22F was inconsistent across the literature.^18^, ^49^, ^50^ The genetic background for serotype 22F isolates was not diverse and mainly associated with CC433 (GPSC19). The other ten most prevalent lineages express non-vaccine serotypes that are not significantly associated with disease (serotypes 6C, 10A, 15A, 15B/C, 21, 22F, 23A, 23B, 35B, and 38; appendix 1).^18^ The invasive disease potential of serotypes is not the only determinant of disease replacement; serotypes with low invasive disease potential can still cause disease among individuals with comorbid conditions. Furthermore, given that nasopharyngeal carriage of pneumococcus could be a risk for proceeding into invasive disease, frequent colonisers, such as serotypes 15B/C and 35B, might be sufficiently prevalent in carriage for expansion to result in significant disease replacement.^51^, ^52^

Despite the overall reductions in prevalence of antibiotic resistance in pneumococci after the use of PCVs, significant increases in penicillin and erythromycin resistance among non-vaccine-type isolates were observed in the PCV13 period. Penicillin is the first-line antibiotic in the treatment of pneumococcal disease. The increasing empirical use of macrolides for pneumonia has been observed in high-income countries, such as the USA, due to their broader spectrum activity against atypical pneumonia caused by *Mycoplasma pneumoniae, Chlamydia pneumoniae*, and *Bordetella pertussis*.^53^, ^54^, ^55^ The increase in penicillin and erythromycin resistance could be due to acquisition of resistance determinants (eg, *mefA* in GPSC3 in the USA), the expansion of extant non-vaccine serotypes within a well established resistant lineage (eg, penicillin-resistant lineage GPSC5 replacing its major serotype composition from serotype 23F to 35B/D in South Africa), or simply a clonal expansion of a non-vaccine-type lineage with antimicrobial resistance (eg, penicillin and macrolide resistant GPSC59 in the USA). Additionally, this study revealed differences in resistance prevalence within a single lineage between countries, indicating that some surviving lineages could acquire antibiotic resistance determinants adaptive to the local antibiotic selective pressure.

A limitation of this study is the relatively short length of sampling time after PCV13 introduction, as indicated by the presence of residual PCV13 serotypes in the top five serotypes in some countries, especially in the USA. This limitation also hinders our ability to account for secular trends. Given this context, the described changes are early indicators of restructuring of the population towards its new equilibrium. Subtler changes, taking longer to manifest, would not have been detected, but the effects of local population structure on serotype replacement is clear. Despite small numbers of samples per year throughout the pre-PCV period in Hong Kong and The Gambia, collections were representative of the baseline population in each country (PMC15308357 and PMC16827713). The small number of samples from Malawi after introduction of PCV13 was the result of the low incidence of pneumooccal disease in that period, rather than a problem of low representation. Increasing surveillance intensity was observed in South Africa during the first 3 years of the pre-PCV period; therefore, we might have underestimated the actual reductions in vaccine serotypes and overestimated the actual increase in non-vaccine serotypes.^2^ However, the surveillance intensity in the last 2 years of the pre-PCV period were similar to the rest of the study periods.

In conclusion, globally spreading lineages expressing invasive serotypes have an important role in serotype replacement, and emerging non-vaccine serotypes associated with different pneumococcal lineages in different countries might be explained by local antibiotic selective pressures. Continued genomic surveillance of the dynamics of pneumococcal populations with increased geographical representation in the post-vaccine period will generate further knowledge for optimising future vaccine design.
