# Supplementary material for: Pneumococcal lineages associated with serotype replacement and antibiotic resistance in childhood invasive pneumococcal disease in the post-PCV13 era: an international whole-genome sequencing study
Source: Lancet Infect Dis. 2019 Jul;19(7):759–69. doi: 10.1016/S1473-3099(19)30297-X (PMC7641901; doi:10.1016/S1473-3099(19)30297-X)
Supplement: Supplementary appendix 1 [file mmc1.pdf]

THE LANCET
Infectious Diseases

Supplementary appendix

This appendix formed part of the original submission and has been peer reviewed. We post it as supplied by the authors.

Supplement to: Lo SW, Gladstone RA, van Tonder AJ, et al, The Global Pneumococcal Sequencing Consortium. Pneumococcal lineages associated with serotype replacement and antibiotic resistance in childhood invasive pneumococcal disease in the post-PCV13 era: an international whole-genome sequencing study. *Lancet Infect Dis* 2019; published online June 10. http://dx.doi.org/10.1016/S1473-3099(19)30297-X.

Appendix 1

Supplement to: Stephanie W. Lo, Rebecca A. Gladstone, Andries van Tonder *et al*. Pneumococcal Lineages Associated with Serotype Replacement and Antibiotic Resistance in Childhood Invasive Pneumococcal Disease in the Post-PCV13 Era: An international whole genome sequencing study

Supplementary Methodology

Bacterial collection in the GPS database

The Global Pneumococcal Sequencing (GPS) database (last accessed on 1st June 2017) consisted of 13,454 pneumococcal isolates from 30 countries, 1991-2016. It included disease (n=8,605) and carriage (n=4,849) isolates, representing 30 countries from Africa (59%, 7,928/13,454), Asia (18%, 2,381/13,454), North America (13%, 1,684/13,454), South America (7%, 1,027/13,454), and Europe (3%, 434/13,454). Of the 8,605 disease isolates, 4,416 (52%) were invasive isolates that recovered from children aged <3 years old from 27 countries, 1991-2016 (Table 1).

Genotypic prediction of antibiotic resistance

Using the overall GPS database (n=13,454), sensitivity, specificity, positive predictive value and negative predictive value of genotypic antimicrobial resistance with phenotypic susceptibility results were all >90%, except for the sensitivity for predicting chloramphenicol resistance (> 75%) (1). Positive predictive value for each genetic resistance determinant were all >95%, except for the positive predictive value of *folP* for the non-susceptibility to cotrimoxazole (72%) (1). In view of the incomplete phenotypic susceptibility testing results and high concordance between phenotype and genotype, we assessed the prevalence antibiotic resistance based on the resistance genotypes throughout the study.

Detection of changes in invasive pneumococcal disease across vaccine periods

Incidence rate ratios (IRRs) for total invasive pneumococcal disease (IPD), non-vaccine serotype (NVT) IPD or NVT IPD within Global Pneumococcal Sequencing Cluster (GPSC) were calculated for South Africa, Israel and USA between the pre-PCV and PCV7 or PCV13 periods with the estimated number of IPD cases per year for each two periods. Either Poisson regression or negative binomial regression was used, depending on the presence of over-dispersion that evaluated by the goodness-of-fit with a chi-square test based on the residual deviance and degrees of freedom. We used the Poisson regression model if there was no over-dispersion (Poisson goodness-of-fit p value >0.05). For mild over-dispersion (Poisson goodness-of-fit p value ranged between 0.01 and 0.05), we used Poisson regression model with robust standard errors for the parameter estimates (2). We calculated the robust standard errors and p values using R package sandwich (3). If over-dispersion was detected (Poisson goodness-of-fit p value <0.01), negative binominal regression was used. We did not observe any zero inflation in our data. The GPSCs with ≤5 isolates were not included for analysis. All of the

models converged according to the glm object; model$converged = TRUE. In both regression models, the dependent variable was the estimated annual IPD cases and the independent variable was the vaccine period for each year (e.g. pre-PCV period, PCV7 period or PCV13 period). As only a random subset of isolates was whole genome sequenced from each country, we estimated the actual number of IPD cases by dividing the number of observed genomes over the proportion of cases randomly selected for whole genome sequencing in each collection year. Cases were rounded to the nearest integer. We used an offset term representing the mid-year estimates of population size.(4) If the data did not fit to both regression models, an average IRR were calculated by dividing the incidence rate in each post-vaccine period by the incidence rate in the pre-PCV period using R package epiR (5). To avoid IRR being calculated as zero or infinity, we added a constant number of one to all the estimated IPD cases for both periods if a GPSC was not observed in one of the two PCV periods. Serotype replacement was detected if the IRR and 95% confidence interval (CI) were above one for South Africa, Israel and USA.(4) The goodness-of-fit, choice of model and statistical output were deposited on github https://github.com/rgladstone/GPSCs/tree/master/lo_et_al.

Detection of changes in antibiotic resistance across vaccine periods using binomial generalised linear models

A binomial generalised linear regression was performed individually for five different antibiotic classes (penicillin, chloramphenicol, cotrimoxazole, erythromycin and tetracycline) and multidrug resistance on either total isolates or NVT isolates. Categorical resistance status ("non-susceptible" or "susceptible") of the isolates was the dependent variable. The independent variables were country of isolation and PCV period. Country was included as dependant variable to account for variations in resistance prevalence between countries. The sign of the PCV period coefficient estimate indicates the direction of change and the PCV period coefficient Pr(>|z|) used to assess significance. The R script used for this analysis was deposited on github https://github.com/rgladstone/GPSCs/tree/master/lo_et_al.

Table 1. The number of pneumococcal isolates recovered from children aged <3 years old with pneumococcal disease from 27 countries, 1991-2016.

Country	Pre-PCV	Post-PCV7	Post-PCV10	Post-PCV13	Total
South Africa[a]	764	299	-	442	1505
USA[b]	173	410	-	127	710
Israel	357	67	-	277	701
Brazil	119	-	112	-	231
Malawi[c]	213	-	-	15	228
The Gambia[c]	62	56	-	102	220
Mozambique	138	-	-	-	138
Poland	121	-	-	-	121
Peru	72	19	-	-	91
Hong Kong, China	45	13	-	20	78
Slovenia	74	-	-	-	74
Bangladesh	73	-	-	-	73
India	53	-	-	-	53
Morocco	22		2	6	30
Qatar	-	11	-	19	30
Nepal	25	-	-	-	25
Trinidad and Tobago	11	-	11	-	22
Senegal	19	-	-	-	19
Togo	16	-	-	-	16
Egypt	14	-	-	-	14
Niger	12	-	-	-	12
Cameroun	-	-	-	8	8
Russian Federation	5	-	-	-	5
Malaysia	4	-	-	-	4
Canada	-	3	-	-	3
Ghana	3	-	-	-	3
Belarus	2	-	-	-	2
Grand Total	2397	878	125	1016	4416

[a]One, two and one hundred and fifty-one invasive pneumococcal isolates from South Africa collected in 1991, 2011 and 2012 were excluded from this study, respectively. The exclusion of the isolates collected in 2012, one year after the PCV13 introduction, from the PCV13 period improved the sensitivity for detection of any serotype replacement.

[b]Thirty-six invasive pneumococcal isolates from the USA collected in 2000 were not included in the pre-PCV period in this study on the basis that the extensive PCV7 vaccination and catch-up programme in the USA might lead to changes in serotype and genotype immediately.

[c] Two and nineteen invasive pneumococcal isolates from Malawi and The Gambia collected in 2012 were not included in the PCV13 period in this study, respectively. The exclusion of the isolates collected one year (2012) from the PCV13 period improved the sensitivity for detecting any increase in invasive pneumococcal disease caused by non-vaccine serotype pneumococci.

Table 2. Changes in invasive pneumococcal disease in incidence between the pre-PCV and PCV13 period in Israel, South Africa and the USA.

	Estimated average incidence rate per year			
Country	Pre-PCV	Post-PCV13	IRR (95% confidence interval)	P value
Israel	69.6	29.3	0.42 (0.34-0.53)	< 0.0001
South Africa	30.7	8.2	0.27 (0.23-0.30)	< 0.0001
USA	144.3	12.0	0.08 (0.07-0.10)	< 0.0001

Table 3. Changes in invasive pneumococcal disease in prevalence between the pre-PCV and PCV13 period in Hong Kong, Malawi and The Gambia.

	Number of isolates (%)				
Country	Pre-PCV VT	Pre-PCV total	PCV13 VT	PCV13 total	P value
Hong Kong	44 (98)	45	15 (75)	20	0.0089
Malawi	160 (75)	213	5 (38)	13	0.0075
The Gambia	56 (90)	62	34 (40)	86	0.0001

Table 4. The serotype distribution of pneumococcal isolates from Hong Kong, Israel, USA, South Africa, The Gambia and Malawi in the PCV13 period

Countries	Serotypes	Number of isolates (%)[a]	Remarks[b]
Hong Kong	3	9 (45.0)	-
(n=20)	19A	4 (20.0)	-
	15B/15C	3 (15.0)	-
	Others	4 (20.0)	14 (1), 15A (1), 19F (1), 23A (1)
Israel	12F	68 (24.5)	-
(n=277)	15B/15C	27 (9.7)	-
	5	26 (9.4)	-
	19A	17 (6.1)	-
	33F	13 (4.7)	-
	1	12 (4.3)	-
	24	12 (4.3)	-
	10A	10 (3.6)	-
	10B	9 (3.2)	-
	38	8 (2.9)	-
	15A	8 (2.9)	-
	16F	7 (2.5)	-
	7F	7 (2.5)	-
	3	4 (1.4)	-
	27	4 (1.4)	-
	22F	4 (1.4)	-
	35B/35D	4 (1.4)	-
	2	3 (1.1)	-
	14	3 (1.1)	-
	23A	3 (1.1)	-
	23B	3 (1.1)	-
	23F	3 (1.1)	-
	6A	3 (1.1)	-
	7B	3 (1.1)	-
	others	16 (5.8)	6B (2), 8 (2), 9V (2), 18C (2), 9N (1), 11A (1), 13 (1), 15F (1), 17F (1), 18A (1), 19F (1), 21 (1)
USA	15B/15C	16 (12.6)	-
(n=127)	22F	16 (12.6)	-
	19A	14 (11.0)	-
	33F	12 (9.4)	-
	35B/35D	11 (8.7)	-
	12F	9 (7.1)	-
	38	7 (5.5)	-
	10A	5 (3.9)	-
	11A	5 (3.9)	-
	15A	4 (3.1)	-
	6C	4 (3.1)	-
	3	3 (2.4)	-

	21	3 (2.4)	-
	23B	3 (2.4)	-
	Others	15 (11.8)	8 (2), 9N (2), 23A (2), 31 (2), 4 (1), 7C (1), 7F (1), 18C (1), 19F (1), 34 (1), 35F (1)
South Africa	8	32 (11.0)	-
(n=291)	35B/35D	23 (7.9)	-
	12F	22 (7.6)	-
	15B/15C	22 (7.6)	-
	16F	19 (6.5)	-
	19A	15 (5.1)	-
	15A	14 (4.8)	-
	23F	13 (4.5)	-
	19F	10 (3.4)	-
	10A	9 (3.1)	-
	17F	9 (3.1)	-
	6A	9 (3.1)	-
	6B	9 (3.1)	-
	21	6 (2.1)	-
	18C	6 (2.1)	-
	1	5 (1.7)	-
	3	5 (1.7)	-
	13	5 (1.7)	-
	23A	5 (1.7)	-
	33D	5 (1.7)	-
	7C	5 (1.7)	-
	34	4 (1.4)	-
	11A	4 (1.4)	-
	9N	4 (1.4)	-
	14	3 (1.0)	-
	others	28 (9.6)	4 (2), 7F (2), 9V (2), 20A (2), 23B (2), 28A (2), 33F (2), 38 (2), 5 (1), 10F (1), 19B (1), 22F (1), 24 (1), 27 (1), 28F (1), 29 (1), 33A (1), 35A (1), 35F (1), NT (1)
The Gambia	12F	20 (23.3)	-
(n=86)	1	13 (15.1)	-
	23F	6 (7.0)	-
	5	5 (5.8)	-
	35B/35D	5 (5.8)	-
	14	4 (4.7)	-
	24	4 (4.7)	-
	11B	4 (4.7)	-
	15B/15C	4 (4.7)	-

	Others	21 (24.4)	2 (2), 10F (2), 13 (2), 18C (2), 33F (2), 3 (1), 6A (1), 16F (1), 17F (1), 19A (1), 19F (1), 21 (1), 25F (1), 38 (1), 40 (1), 46 (1)
Malawi	35B/35D	4 (30.8)	-
	Others	9 (69.2)	1 (1), 2 (1), 5 (1), 7F (1), 12F (1), 13 (1), 18C (1), 21 (1), 23F (1)

[a]Percentage may not sum up to 100% due to rounding.
[b]The serotypes with ≤2 isolates were listed. The number of each serotype was indicated in brackets.

Table 5. The serotype distribution of pneumococcal isolates from Hong Kong by vaccine periods

Vaccine periods	Serotypes	Number of isolates (%)[a]	Remarks[b]
Pre-PCV period	14	16 (35.6)	-
(n=45)	6B	13 (28.9)	-
	23F	6 (13.3)	-
	19F	4 (8.9)	-
	Others	6 (13.3)	9V (2), 18C (2), 2 (1), 3 (1)
PCV7 period	3	5 (38.5)	-
(n=13)	19A	4 (30.8)	-
	others	4 (30.8)	6B (1), 14 (1), 15B/15C (1), 19F (1)
PCV13 period	3	9 (45.0)	-
(n=20)	19A	4 (20.0)	-
	15B/15C	3 (15.0)	-
	Others	4 (20.0)	14 (1), 15A (1), 19F (1), 23A (1)

[a]Percentage may not sum up to 100% due to rounding.
[b]The serotypes with ≤2 isolates were listed. The number of each serotype was indicated in brackets.

Table 6. The serotype distribution of pneumococcal isolates from Israel by vaccine periods

Vaccine periods/years	Serotypes	Number of isolates (%)[a]	Remarks[b]
Pre-PCV period	14	49 (13.7)	-
(n=357)	6B	43 (12.0)	-
	19A	36 (10.1)	-
	6A	31 (8.7)	-
	1	30 (8.4)	-
	23F	28 (7.8)	-
	9V	27 (7.6)	-
	5	21 (5.9)	-
	19F	18 (5.0)	-
	18C	12 (3.4)	-
	12F	9 (2.5)	-
	33F	8 (2.2)	-
	4	5 (1.4)	-
	8	4 (1.1)	-
	46	4 (1.1)	-
	15A	4 (1.1)	-
	24	3 (0.8)	-
	38	3 (0.8)	-
	15B/15C	3 (0.8)	-
	Others	19 (5.3)	13 (2), 23B (2), 27 (2), 28A (2), 34 (2), 2 (1), 7F (1), 10B (1), 11A (1), 18A (1), 18B (1), 22A (1), 23A (1), 35B/35D (1)
PCV7 period	1	18 (26.9)	-
(n=67)	19A	9 (13.4)	-
	5	8 (11.9)	-
	3	3 (4.5)	-
	12F	3 (4.5)	-
	15B/15C	3 (4.5)	-
	19F	3 (4.5)	-
	Others	20 (29.9)	6B (2), 6C (2), 18C (2), 38 (2), 4 (1), 7F (1), 8 (1), 9N (1), 11A (1), 11B (1), 15A (1), 22F (1), 23F (1), 27 (1), 33F (1), 35B/35D (1)
PCV13 period 2011	5	18 (21.7)	-
(n=83)	1	9 (10.8)	-
	12F	8 (9.6)	-
	19A	8 (9.6)	-
	15B/15C	5 (6.0)	-
	7F	5 (6.0)	-
	24	4 (4.8)	-
	10A	4 (4.8)	-
	22F	3 (3.6)	-

	33F	3 (3.6)	-
	others	16 (19.3)	6A (2), 6B (2), 10B (2), 38 (2), 3 (1), 9V (1), 11A (1), 14 (1), 16F (1), 23F (1), 27 (1), 35B/35D (1)
PCV13 period 2012	12F	22 (31.9)	-
(n=69)	15B/15C	7 (10.1)	-
	5	5 (7.2)	-
	19A	4 (5.8)	-
	24	3 (4.3)	-
	10B	3 (4.3)	-
	33F	3 (4.3)	-
	others	22 (31.9)	1 (2), 3 (2), 10A (2), 15A (2), 23A (2), 23F (2), 27 (2), 38 (2), 2 (1), 16F (1), 17F (1), 18A (1), 21 (1), 35B/35D (1)
PCV13 period 2014	12F	16 (33.3)	-
(n=48)	15B/15C	7 (14.6)	-
	24	3 (6.3)	-
	19A	3 (6.3)	-
	7B	3 (6.3)	-
	Others	16 (33.3)	5 (2), 16F (2), 33F (2), 3 (1), 6A (1), 8 (1), 9N (1), 10A (1), 10B (1), 14 (1), 15A (1), 19F (1), 23A (1)

[a]Percentage may not sum up to 100% due to rounding.

[b]The serotypes with ≤2 isolates were listed. The number of each serotype was indicated in brackets.

Table 7. The serotype distribution of pneumococcal isolates from Malawi by vaccine periods

Vaccine periods/years	Serotypes	Number of isolates (%)[a]	Remarks[b]
Pre-PCV period	5	27 (12.7)	-
(n=213)	23F	23 (10.8)	-
	6B	23 (10.8)	-
	6A	18 (8.5)	-
	1	17 (8.0)	-
	19A	11 (5.2)	-
	4	10 (4.7)	-
	19F	9 (4.2)	-
	14	7 (3.3)	-
	35B/35D	7 (3.3)	-
	15B/15C	6 (2.8)	-
	13	5 (2.3)	-
	18C	5 (2.3)	-
	9V	5 (2.3)	-
	12F	4 (1.9)	-
	16F	4 (1.9)	-
	7F	4 (1.9)	-
	12B	3 (1.4)	-
	9L	3 (1.4)	-
	Others	22 (10.3)	2 (2), 8 (2), 10B (2), 23A (2), 29 (2), 3 (1), 7C (1), 10A (1), 15A (1), 18F (1), 19B (1), 20B (1), 24 (1), 33D (1), 33F (1), 34 (1), 38 (1)
PCV13 period	35B/35D	4 (30.8)	-
(n=13)	Others	9 (69.2)	1 (1), 2 (1), 5 (1), 7F (1), 12F (1), 13 (1), 18C (1), 21 (1), 23F (1)

[a]Percentage may not sum up to 100% due to rounding.
[b]The serotypes with ≤2 isolates were listed. The number of each serotype was indicated in brackets.

Table 8. The serotype distribution of pneumococcal isolates from South Africa by vaccine periods

Vaccine periods/years	Serotypes	Number of isolates (%)[a]	Remarks[b]
Pre-PCV period	14	112 (14.7)	-
(n=763)	6B	95 (12.5)	-
	6A	91 (11.9)	-
	23F	90 (11.8)	-
	19F	72 (9.4)	-
	19A	62 (8.1)	-
	1	42 (5.5)	-
	18C	28 (3.7)	-
	8	16 (2.1)	-
	15B/15C	16 (2.1)	-
	9V	13 (1.7)	-
	3	12 (1.6)	-
	4	12 (1.6)	-
	12F	11 (1.4)	-
	5	7 (0.9)	-
	29	7 (0.9)	-
	22F	6 (0.8)	-
	34	5 (0.7)	-
	15A	5 (0.7)	-
	16F	5 (0.7)	-
	7F	5 (0.7)	-
	10A	4 (0.5)	-
	23A	4 (0.5)	-
	7C	4 (0.5)	-
	9N	4 (0.5)	-
	13	3 (0.4)	-
	11A	3 (0.4)	-
	23B	3 (0.4)	-
	33D	3 (0.4)	-
	Others	23 (3.0)	17F (2), 18A (2), 18B (2), 18F (2), 20A (2), 21 (2), 25F (2), 35A (2), 38 (2), 6C (1), 9L (1), 10X (1), 31 (1), 35F (1)
Post-PCV7 period	19A	43 (14.5)	-
(n=297)	6A	36 (12.1)	-
	14	33 (11.1)	-
	19F	26 (8.8)	-
	23F	25 (8.4)	-
	6B	24 (8.1)	-
	1	15 (5.1)	-
	5	10 (3.4)	-
	12F	9 (3.0)	-
	15B/15C	9 (3.0)	-
	8	8 (2.7)	-

	9V	7 (2.4)	-
	16F	6 (2.0)	-
	17F	5 (1.7)	-
	38	4 (1.3)	-
	22F	4 (1.3)	-
	3	3 (1.0)	-
	4	3 (1.0)	-
	13	3 (1.0)	-
	18C	3 (1.0)	-
	33D	3 (1.0)	-
	35B/35D	3 (1.0)	-
	Others	15 (5.1)	9N (2), 29 (2), 33F (2), 6C (1), 7C (1), 10A (1), 10X (1), 15A (1), 20A (1), 23A (1), 23B (1), 33A (1)
Post-PCV13 period 2013	8	15 (10.6)	-
(n=141)	15B/15C	12 (8.5)	-
	12F	10 (7.1)	-
	35B/35D	10 (7.1)	-
	16F	8 (5.7)	-
	23F	8 (5.7)	-
	15A	7 (5.0)	-
	19A	6 (4.3)	-
	10A	5 (3.5)	-
	17F	5 (3.5)	-
	19F	5 (3.5)	-
	6A	5 (3.5)	-
	18C	4 (2.8)	-
	6B	4 (2.8)	-
	3	3 (2.1)	-
	21	3 (2.1)	-
	34	3 (2.1)	-
	23A	3 (2.1)	-
	Others	25 (17.7)	1 (2), 9V (2), 11A (2), 20A (2), 28A (2), 33D (2), 4 (1), 7F (1), 10F (1), 13 (1), 14 (1), 22F (1), 23B (1), 27 (1), 29 (1), 33F (1), 35A (1), 35F (1), 38 (1)
Post-PCV13 period 2014	8	17 (11.3)	-
(n=150)	35B/35D	13 (8.7)	-
	12F	12 (8.0)	-
	16F	11 (7.3)	-
	15B/15C	10 (6.7)	-
	19A	9 (6.0)	-
	15A	7 (4.7)	-
	19F	5 (3.3)	-

	23F	5 (3.3)	-
	6B	5 (3.3)	-
	7C	5 (3.3)	-
	13	4 (2.7)	-
	10A	4 (2.7)	-
	17F	4 (2.7)	-
	6A	4 (2.7)	-
	9N	4 (2.7)	-
	1	3 (2.0)	-
	21	3 (2.0)	-
	33D	3 (2.0)	-
	Others	22 (14.7)	3 (2), 11A (2), 14 (2), 18C (2), 23A (2), 7F (1), 19B (1), 23B (1), 24 (1), 28F (1), 33A (1), 33F (1), 34 (1), 38 (1), NT (1)

[a]Percentage may not sum up to 100% due to rounding.
[b]The serotypes with ≤2 isolates were listed. The number of each serotype was indicated in brackets.

Table 9. The serotype distribution of pneumococcal isolates from the USA by vaccine periods

Vaccine periods	Serotypes	Number of isolates (%)[a]	Remarks[b]
Pre-PCV period	14	48 (35.0)	-
(n=137)	18C	14 (10.2)	-
	19F	12 (8.8)	-
	4	11 (8.0)	-
	6A	10 (7.3)	-
	6B	9 (6.6)	-
	9V	9 (6.6)	-
	23F	6 (4.4)	-
	22F	3 (2.2)	-
	Others	15 (10.9)	1 (2), 19A (2), 3 (1), 5 (1), 7F (1), 9N (1), 10A (1), 12F (1), 15B/15C (1), 16F (1), 18B (1), 24 (1), 33F (1)
PCV7 period	19A	162 (39.5)	-
(n=410)	7F	56 (13.7)	-
	22F	22 (5.4)	-
	33F	21 (5.1)	-
	15B/15C	18 (4.4)	-
	3	15 (3.7)	-
	6C	11 (2.7)	-
	38	10 (2.4)	-
	10A	10 (2.4)	-
	12F	9 (2.2)	-
	35B/35D	8 (2.0)	-
	19F	7 (1.7)	-
	14	6 (1.5)	-
	15A	6 (1.5)	-
	16F	6 (1.5)	-
	11A	5 (1.2)	-
	23B	5 (1.2)	-
	8	4 (1.0)	-
	6B	4 (1.0)	-
	4	3 (0.7)	-
	17F	3 (0.7)	-
	18C	3 (0.7)	-
	Others	16 (3.9)	1 (2), 5 (2), 9V (2), 21 (2), 35F (2), 6A (1), 9N (1), 18B (1), 23F (1), 24 (1), 34 (1)
PCV13 period	15B/15C	16 (12.6)	-
(n=127)	22F	16 (12.6)	-
	19A	14 (11.0)	-
	33F	12 (9.4)	-
	35B/35D	11 (8.7)	-
	12F	9 (7.1)	-
	38	7 (5.5)	-

	10A	5 (3.9)	-
	11A	5 (3.9)	-
	15A	4 (3.1)	-
	6C	4 (3.1)	-
	3	3 (2.4)	-
	21	3 (2.4)	-
	23B	3 (2.4)	-
	Others	15 (11.8)	8 (2), 9N (2), 23A (2), 31 (2), 4 (1), 7C (1), 7F (1), 18C (1), 19F (1), 34 (1), 35F (1)

[a]Percentage may not sum up to 100% due to rounding.
[b]The serotypes with ≤2 isolates were listed. The number of each serotype was indicated in brackets.

Table 10. The serotype distribution of pneumococcal isolates from The Gambia by vaccine periods

Vaccine periods	Serotypes	Number of isolates (%)[a]	Remarks[b]
Pre-PCV period	1	15 (24.2)	-
(n=62)	5	11 (17.7)	-
	14	9 (14.5)	-
	23F	7 (11.3)	-
	6B	3 (4.8)	-
	Others	17 (27.4)	2 (2), 7F (2), 9V (2), 19A (2), 19F (2), 3 (1), 6A (1) 16F (1), 18C (1), 22F (1), 35B/35D (1), 46 (1)
PCV7 period	5	13 (23.6)	-
(n=55)	1	12 (21.8)	-
	12F	7 (12.7)	-
	6B	3 (5.5)	-
	Others	20 (36.4)	7B (2), 14 (2), 19F (2), 20A (2), 23F (2), 4 (1), 6A (1), 9L (1), 9V (1), 10A (1), 11A (1), 15A (1), 19A (1), 40 (1), 46 (1)
PCV13 period 2013	12F	12 (28.6)	-
(n=42)	1	8 (19.0)	-
	23F	6 (14.3)	-
	5	3 (7.1)	-
	Others	13 (31.0)	14 (2), 2 (1), 3 (1), 6A (1), 13 (1), 15B/15C (1), 16F (1), 19A (1), 19F (1), 24 (1), 35B/35D (1), 40 (1)
PCV13 period 2014	12F	8 (18.2)	-
(n=44)	1	5 (11.4)	-
	11B	4 (9.1)	-
	35B/35D	4 (9.1)	-
	24	3 (6.8)	-
	15B/15C	3 (6.8)	-
	Others	17 (38.6)	5 (2), 10F (2), 14 (2), 18C (2), 33F (2), 2 (1), 13 (1), 17F (1), 21 (1), 25F (1), 38 (1), 46 (1)

[a]Percentage may not sum up to 100% due to rounding.

[b]The serotypes with ≤2 isolates were listed. The number of each serotype was indicated in brackets.

Table 11. The changes in annual incidence rate in Israel between the pre-PCV and PCV7 period

GPSC	Method	Annual incidence rate in the Pre-PCV period	Annual incidence rate in the PCV7 period	IRR (95% confidence interval)	Adjusted p value
1	negative binomial	0.9	1.3	1.34 (0.14-43.36)	0.8727
2	average IRR	3.9	6.7	1.76 (0.95-3.38)	0.1060
3	Poisson model	2.0	1.3	0.62 (0.24-1.35)	0.0021
5	Poisson model	4.9	2.5	0.52 (0.27-0.90)	0.1193
6	negative binomial	10.5	0.2	0.02 (0-0.11)	0.0018*
7	negative binomial	2.4	0.2	0.09 (0-1.05)	0.1733
8	negative binomial	5.0	4.8	0.96 (0.27-4.96)	0.9626
10	Poisson robust SE	1.0	0.6	0.64 (0.38-1.08)	0.2465
11	average IRR	0.8	4.8	5.37 (1.84-21.37)	0.0031*
13	Poisson model	4.3	0.2	0.05 (0-0.22)	0.0141*
16	negative binomial	0.6	0.6	1.09 (0.05-298.98)	0.9626
18	negative binomial	1.4	0.2	0.15 (0.01-1.22)	0.2741
23	negative binomial	2.0	0.2	0.11 (0-2.84)	0.3007
26	negative binomial	1.3	0.2	0.16 (0.01-2.01)	0.3146
29	negative binomial	0.8	1.3	1.63 (0.21-31.11)	0.7508
31	negative binomial	1.4	4.2	3.11 (0.78-18.69)	0.3007
38	negative binomial	0.4	1.3	3.14 (0.28-163.39)	0.5458
43	Poisson model	2.2	0.2	0.10 (0.01-0.43)	0.0837
44	Poisson model	0.8	0.6	0.82 (0.19-2.45)	0.8234
47	negative binomial	5.3	1.3	0.24 (0.06-1.12)	0.1646
50	negative binomial	0.7	1.3	1.86 (0.26-27.64)	0.6459
55	negative binomial	0.8	1.9	2.36 (0.32-45.65)	0.5640
67	Poisson robust SE	2.0	0.2	0.10 (0.07-0.16)	<0.0001*
85	negative binomial	1.6	0.2	0.13 (0.01-2.17)	0.3007
113	Poisson model	2.5	0.2	0.08 (0-0.39)	0.0640
185	negative binomial	1.2	0.2	0.18 (0.01-2.16)	0.3415
198	Poisson model	0.8	0.6	0.82 (019-2.45)	0.8234

GPSC, Global Pneumococcal Sequence Cluster.
P value < 0.05 was flagged with asterisk.

Table 12. The changes in annual incidence rate in Israel between the pre-PCV and PCV13 period

GPSC	Method	Annual incidence rate in the Pre-PCV period	Annual incidence rate in the PCV7 period	IRR (95% confidence interval)	Adjusted p value
1	negative binomial	0.9	0.1	0.10 (0.01-1.14)	0.0969
2	negative binomial	4.0	0.5	0.11 (0.02-0.67)	0.0243*
3	Poisson model	2.0	1.4	0.69 (0.42-1.09)	0.1803
5	Poisson robust SE	4.9	1.2	0.26 (0.14-0.48)	0.0001*
6	negative binomial	10.2	0.4	0.04 (0.02-0.09)	<0.0001*
7	negative binomial	2.1	0.5	0.26 (0.08-0.79)	0.0354*
8	negative binomial	5.0	3.0	0.60 (0.19-2.04)	0.4985
9	negative binomial	0.4	0.5	1.53 (0.04-82.71)	0.8391
10	negative binomial	1.0	0.5	0.55 (0.20-1.51)	0.3493
11	negative binomial	0.8	2.9	3.66 (0.94-15.20)	0.0999
13	Poisson robust SE	4.1	0.1	0.02 (0-0.14)	0.0001*
16	negative binomial	0.6	0.8	1.38 (0.20-10.57)	0.8073
18	Poisson robust SE	1.4	0.2	0.15 (0.08-0.27)	<0.0001*
23	negative binomial	1.7	0.1	0.06 (0-0.99)	0.0621
26	negative binomial	1.1	0.3	0.32 (0.06-1.63)	0.2287
27	negative binomial	0.5	0.1	0.19 (0.01-4.75)	0.3554
29	negative binomial	0.8	0.1	0.13 (0.01-1.25)	0.1194
31	negative binomial	1.4	0.9	0.68 (0.11-4.6)	0.7370
32	average IRR	0.1	0.6	2.65 (0.21-139.12)	0.4639
35	Poisson model	0.2	0.7	3.09 (1.18-9.57)	0.0581
38	negative binomial	0.4	0.7	1.87 (0.34-11.11)	0.5694
43	Poisson model	2.2	0.2	0.09 (0.03-0.22)	<0.0001*
44	Poisson robust SE	0.8	0.2	0.32 (0.11-1.00)	0.0848
47	negative binomial	5.3	0.1	0.02 (0-0.08)	<0.0001*
50	negative binomial	0.7	0.1	0.22 (0.02-2.14)	0.2531
55	average IRR	0.8	6.5	7.29 (2.59-28.32)	<0.0001*
67	Poisson model	2.0	0.2	0.10 (0.03-0.24)	<0.0001*
85	negative binomial	1.4	0.1	0.07 (0.01-0.95)	0.0648
113	Poisson model	2.5	0.2	0.08 (0.02-0.20)	<0.0001*
139	Poisson model	0.1	0.9	6.63 (2.24-28.28)	0.0060*
156	Poisson model	0.2	0.7	3.31 (1.28-10.19)	0.0407*
185	negative binomial	1.2	0.2	0.17 (0.04-0.60)	0.0189*
226	Poisson robust SE	0.3	0.3	1.10 (0.32-3.87)	0.9058
262	average IRR	0.6	0.2	0.29 (0.01-3.67)	0.3494

GPSC, Global Pneumococcal Sequence Cluster.
P value < 0.05 was flagged with asterisk.

Table 13. The changes in annual incidence rate in South Africa between the pre-PCV and PCV7 period

GPSC	Method	Annual incidence rate in the Pre-PCV period	Annual incidence rate in the PCV7 period	IRR (95% confidence interval)	Adjusted p value
1	negative binomial	1.1	0.4	0.36 (0.20-0.65)	0.0039*
2	negative binomial	1.7	0.7	0.39 (0.24-0.65)	0.0018*
3	negative binomial	0.9	0.6	0.65 (0.34-1.28)	0.3661
5	negative binomial	1.3	0.4	0.32 (0.10-1.13)	0.1688
7	average IRR	0.2	0.1	0.39 (0.04-2.38)	0.3443
8	average IRR	0.3	0.4	1.41 (0.56-3.73)	0.5028
9	negative binomial	0.7	0.4	0.59 (0.24-1.54)	0.4228
10	Poisson model	2.3	0.9	0.39 (0.30-0.51)	<0.0001*
11	negative binomial	0.5	0.0	0.10 (0.01-1.35)	0.1646
13	negative binomial	1.7	0.7	0.40 (0.21-0.78)	0.0257*
14	Poisson model	2.5	0.8	0.34 (0.25-0.44)	<0.0001*
17	negative binomial	2.3	1.9	0.82 (0.58-1.15)	0.4157
18	negative binomial	1.3	0.4	0.31 (0.10-1.12)	0.1662
21	negative binomial	1.0	0.6	0.62 (0.25-1.66)	0.4632
22	Poisson robust SE	0.2	0.2	0.94 (0.46-1.92)	0.8993
24	Poisson robust SE	0.7	0.3	0.44 (0.31-0.63)	0.0001*
25	Poisson model	0.0	0.3	8.77 (3.5-26.55)	0.0002*
26	average IRR	0.2	0.2	1.17 (0.30-4.84)	0.8650
30	average IRR	0.1	0.2	1.70 (0.43-7.94)	0.4627
33	negative binomial	0.1	0.2	2.03 (0.27-25.15)	0.5939
37	negative binomial	1.4	0.6	0.40 (0.14-1.29)	0.2465
38	negative binomial	0.1	0.2	2.26 (0.30-28.84)	0.5640
41	negative binomial	1.5	1.2	0.75 (0.44-1.30)	0.4489
48	negative binomial	0.5	0.1	0.23 (0.07-0.75)	0.0640
51	negative binomial	0.4	0.1	0.21 (0.05-0.82)	0.0940
52	Poisson robust SE	0.3	0.2	0.57 (0.30-1.10)	0.2465
54	average IRR	0.4	0.2	0.35 (0.10-1.02)	0.0693
56	negative binomial	0.4	0.2	0.52 (0.13-2.63)	0.5187
67	Poisson model	0.4	0.0	0.12 (0.03-0.32)	0.0020*
68	negative binomial	0.4	0.1	0.14 (0.04-0.46)	0.0113*
77	negative binomial	1.0	0.0	0.05 (0.01-0.18)	0.0002*
79	negative binomial	0.3	0.2	0.55 (0.15-2.29)	0.5187
90	negative binomial	0.4	0.2	0.54 (0.11-3.61)	0.5711
145	average IRR	0.2	0.1	0.49 (0.08-2.28)	0.3865
205	negative binomial	0.3	0.1	0.21 (0.03-2.20)	0.3007

GPSC, Global Pneumococcal Sequence Cluster.
P value < 0.05 was flagged with asterisk.

Table 14. The changes in annual incidence rate in South Africa between the pre-PCV period and PCV13 period

GPSC	Method	Annual incidence rate in the Pre-PCV period	Annual incidence rate in the PCV7 period	IRR (95% confidence interval)	Adjusted p value
1	negative binomial	1.1	0.1	0.12 (0.05-0.25)	<0.0001*
2	negative binomial	1.7	0.1	0.08 (0.04-0.16)	<0.0001*
3	negative binomial	0.9	1.1	1.24 (0.70-2.27)	0.5694
5	Poisson	1.3	0.6	0.46 (0.33-0.64)	<0.0001*
7	average IRR	0.2	0.1	0.56 (0.09-2.88)	0.4996
8	average IRR	0.3	0.0	0.10 (0-0.75)	0.0194*
9	negative binomial	0.7	0.1	0.09 (0.02-0.30)	0.0006*
10	Poisson	2.3	0.2	0.07 (0.04-0.12)	<0.0001*
11	negative binomial	0.5	0.2	0.43 (0.09-3.01)	0.4439
13	negative binomial	1.7	0.2	0.13 (0.06-0.28)	<0.0001*
14	Poisson	2.5	0.3	0.14 (0.09-0.20)	<0.0001*
16	negative binomial	0.1	0.1	1.08 (0.04-198.64)	0.9649
17	negative binomial	2.3	0.4	0.17 (0.10-0.27)	<0.0001*
18	Poisson	1.4	0.0	0.02 (0-0.7)	<0.0001*
21	negative binomial	1.0	0.1	0.11 (0.04-0.27)	<0.0001*
22	negative binomial	0.2	0.2	1.18 (0.43-3.29)	0.8073
24	Poisson robust SE	0.7	0.1	0.09 (0.07-0.12)	<0.0001*
25	Poisson	0.0	0.4	11.19 (4.64-33.23)	<0.0001*
26	negative binomial	0.2	0.3	1.71 (0.35-11.52)	0.6148
30	negative binomial	0.1	0.2	1.86 (0.47-8.99)	0.4985
32	negative binomial	0.1	0.1	0.52 (0.04-13.73)	0.7001
33	negative binomial	0.1	0.3	3.48 (0.56-33.96)	0.2965
34	negative binomial	0.2	0.1	0.76 (0.12-6.80)	0.8272
36	negative binomial	0.0	0.2	3.70 (0.25-167.52)	0.4800
37	negative binomial	1.4	0.1	0.05 (0.02-0.15)	<0.0001*
41	negative binomial	1.6	0.0	0.02 (0-0.06)	<0.0001*
48	negative binomial	0.5	0.2	0.41 (0.14-1.22)	0.1480
49	negative binomial	0.0	0.1	2.33 (0.16-94.81)	0.6389
51	negative binomial	0.4	0.0	0.08 (0.01-0.38)	0.0075*
52	Poisson robust SE	0.3	0.1	0.32 (0.16-0.65)	0.0045*
54	negative binomial	0.4	0.1	0.14 (0.02-1.18)	0.0760
56	negative binomial	0.4	0.3	0.91 (0.25-4.09)	0.9171
67	Poisson	0.4	0.1	0.30 (0.13-0.59)	0.0037*
68	negative binomial	0.4	0.1	0.14 (0.04-0.41)	0.0025*
70	negative binomial	0.1	0.1	0.52 (0.04-13.73)	0.7001
77	negative binomial	1.0	0.1	0.09 (0.03-0.27)	0.0001*
79	negative binomial	0.3	0.0	0.10 (0.01-0.46)	0.0158*
90	negative binomial	0.4	0.0	0.07 (0.01-0.82)	0.0448*

93	average IRR	0.1	0.1	0.93 (0.12-6.96)	0.9317
114	negative binomial	0.1	0.1	1.26 (0.16-17.14)	0.8708
125	average IRR	0.1	0.0	0.47 (0.04-3.25)	0.4595
145	average IRR	0.2	0.0	0.16 (0-1.28)	0.0907
168	Poisson	0.0	0.2	5.13 (1.87-16.28)	0.0060*
205	negative binomial	0.3	0.0	0.09 (0.01-0.57)	0.0254*

GPSC, Global Pneumococcal Sequence Cluster.
P value < 0.05 was flagged with asterisk.

Table 15. The changes in annual incidence rate in the USA between the pre-PCV period and PCV7 period

GPSC	Method	Annual incidence rate in the Pre-PCV period	Annual incidence rate in the PCV7 period	IRR (95% confidence interval)	Adjusted p value
1	average IRR	4.8	3.5	0.70 (0.44-1.13)	0.2093
3	average IRR	2.6	2.2	0.82 (0.44-1.53)	0.5507
4	negative binomial	2.5	6.4	2.51 (0.62-7.61)	0.3007
5	negative binomial	0.1	0.5	3.44 (0.23-37.43)	0.4495
6	negative binomial	9.3	0.7	0.08 (0-1.65)	0.3282
7	Poisson model	4.8	0.0	0.01 (0-0.02)	<0.0001*
9	average IRR	0.7	1.0	1.57 (0.53-5.63)	0.4627
10	negative binomial	0.7	0.0	0.06 (0-4.08)	0.4290
11	negative binomial	1.3	0.3	0.19 (0-14.83)	0.5973
12	average IRR	0.7	1.0	1.45 (0.48-5.25)	0.5507
15	average IRR	0.7	1.7	2.42 (0.88-8.24)	0.1256
16	negative binomial	7.4	1.1	0.14 (0-2.19)	0.4157
18	negative binomial	32.9	0.6	0.02 (0-2.91)	0.3336
19	average IRR	2.0	2.1	1.05 (0.53-2.13)	0.9527
23	negative binomial	8.1	0.7	0.09 (0-15.91)	0.5458
24	average IRR	0.7	0.6	0.97 (0.28-3.75)	0.9630
27	average IRR	11.5	1.5	0.13 (0.07-0.21)	<0.0001*
32	average IRR	0.7	1.0	1.45 (0.48-5.25)	0.5507
35	negative binomial	0.1	0.4	3.30 (0.21-36.81)	0.4684
36	average IRR	2.8	0.6	0.20 (0.07-0.49)	0.0003*
38	negative binomial	0.1	0.9	6.84 (0.50-72.58)	0.2550
39	negative binomial	24.8	0.6	0.02 (0-3.80)	0.3695
50	negative binomial	10.9	1.1	0.10 (0-6.51)	0.5118
97	average IRR	2.0	0.3	0.16 (0.04-0.51)	0.0010*
119	average IRR	1.3	0.8	0.60 (0.23-1.62)	0.3554
135	average IRR	0.7	0.3	0.36 (0.06-1.86)	0.2450

GPSC, Global Pneumococcal Sequence Cluster.
P value < 0.05 was flagged with asterisk.

Table 16. The changes in annual incidence rate in the USA between the pre-PCV and PCV13 period

GPSC	Method	Annual incidence rate in the Pre-PCV period	Annual incidence rate in the PCV7 period	IRR (95% confidence interval)	Adjusted p value
1	Poisson	4.8	0.6	0.12 (0.05-0.24)	<0.0001*
3	average IRR	2.6	1.7	0.62 (0.32-1.21)	0.1973
4	average IRR	2.6	1.4	0.50 (0.25-1.01)	0.0705
6	negative binomial	9.3	0.5	0.05 (0.01-0.18)	<0.0001*
7	Poisson	4.8	0.2	0.03 (0.01-0.11)	<0.0001*
16	Poisson robust SE	7.5	0.2	0.02 (0.02-0.03)	<0.0001*
18	negative binomial	32.9	0.1	0.002 (0-0.03)	<0.0001*
19	average IRR	2.0	1.6	0.79 (0.38-1.66)	0.5656
23	negative binomial	8.2	0.1	0.01 (0-0.04)	<0.0001*
27	Poisson	11.5	0.1	0.01 (0-0.03)	<0.0001*
32	average IRR	0.7	1.0	1.42 (0.47-5.15)	0.5746
38	Poisson	0.1	0.7	5.33 (1.37-34.95)	0.0608
39	negative binomial	24.9	0.1	0.003 (0-0.01)	<0.0001*
50	Poisson	11.1	0.1	0.01 (0-0.03)	<0.0001*
59	Poisson	0.1	1.1	8.29 (2.32-52.78)	0.0121*
132	Poisson	0.1	0.6	4.14 (1.00-27.81)	0.1194

GPSC, Global Pneumococcal Sequence Cluster.
P value < 0.05 was flagged with asterisk.

Table 17. Prevalence of antibiotic resistance by pneumococcal lineage in Israel

		Number of isolates (%)					
GPSC[a]	n	Penicillin	Chloramphenicol	Erythromycin-*ermB*	Erythromycin-*mefA*	Cotrimxoazole	Tetracycline
1	9	8 (88.9)	0	2 (22.2)	8 (88.9)	8 (88.9)	8 (88.9)
2	37	0	0	0	0	37 (100.0)	0
3	27	10 (37.0)	0	2 (7.4)	0	3 (11.1)	2 (7.4)
5	40	40 (100.0)	0	0	6 (15.0)	32 (80.0)	0
6	54	43 (79.6)	0	5 (9.3)	3 (5.6)	52 (96.3)	3 (5.6)
7	17	0	0	0	0	4 (23.5)	0
8	55	0	0	0	0	55 (100.0)	0
9	9	9 (100.0)	0	9 (100.0)	0	0	9 (100.0)
10	12	10 (83.3)	0	10 (83.3)	0	3 (25.0)	12 (100.0)
11	41	13 (31.7)	0	0	0	27 (65.9)	0
13	24	24 (100.0)	0	0	24 (100.0)	0	0
16	12	1 (8.3)	1 (8.3)	0	1 (8.3)	1 (8.3)	2 (16.7)
18	6	0	0	0	6 (100.0)	0	0
23	9	8 (88.9)	6 (66.7)	7 (77.8)	1 (11.1)	8 (88.9)	7 (77.8)
26	10	1 (10.0)	8 (80.0)	1 (10.0)	0	10 (100.0)	10 (100.0)
27	5	3 (60.0)	0	0	0	0	0
29	7	0	0	0	0	0	0
31	23	0	0	0	0	5 (21.7)	0
32	6	0	0	0	0	1 (16.7)	0
35	5	0	0	1 (20.0)	0	0	1 (20.0)
38	13	2 (15.4)	0	0	2 (15.4)	0	3 (23.1)
43	9	0	0	0	6 (66.7)	9 (100.0)	6 (66.7)
44	7	4 (57.1)	0	4 (57.1)	0	0	4 (57.1)
47	26	26 (100.0)	0	26 (100.0)	0	25 (96.2)	26 (100.0)
50	8	0	0	0	0	3 (37.5)	0
55	74	74 (100.0)	0	0	0	0	0
67	8	0	0	0	0	3 (37.5)	3 (37.5)
85	6	0	0	0	0	6 (100.0)	0
113	11	11 (100.0)	0	0	0	11 (100.0)	0
139	10	0	0	0	0	0	10 (100.0)
156	5	5 (100.0)	0	0	0	5 (100.0)	0
185	5	5 (100.0)	0	0	0	4 (80.0)	0
198	5	1 (20.0)	1 (20.0)	1 (20.0)	0	1 (20.0)	1 (20.0)
226	7	0	0	0	0	0	0

262	5	0	0	0	0	0	0
Others[b]	94	31 (33.0)	0	3 (3.2)	3 (3.2)	17 (18.1)	8 (8.5)

[a]GPSC, Global Pneumococcal Sequence Cluster.
[b]Lineages with <5 isolates were not listed individually.

Table 18. Prevalence of antibiotic resistance by pneumococcal lineage in South Africa

		Number of isolates (%)					
GPSC[a]	n	Penicillin	Chloramphenicol	Erythromycin-*ermB*	Erythromycin-*mefA*	Cotrimxoazole	Tetracycline
1	41	40 (97.6)	0	27 (65.9)	27 (65.9)	41 (100.0)	27 (65.9)
2	62	0	2 (3.2)	0	0	3 (4.8)	2 (3.2)
3	72	0	0	1 (1.4)	0	4 (5.6)	1 (1.4)
5	61	59 (96.7)	0	2 (3.3)	8 (13.1)	60 (98.4)	2 (3.3)
7	8	1 (12.5)	0	0	0	2 (25.0)	0
8	18	1 (5.6)	0	0	0	8 (44.4)	0
9	27	26 (96.3)	4 (14.8)	3 (11.1)	0	25 (92.6)	26 (96.3)
10	81	80 (98.8)	0	69 (85.2)	0	80 (98.8)	80 (98.8)
11	19	0	0	0	0	13 (68.4)	0
13	64	13 (20.3)	0	0	2 (3.1)	43 (67.2)	0
14	92	65 (70.7)	17 (18.5)	28 (30.4)	65 (70.7)	86 (93.5)	91 (98.9)
16	6	4 (66.7)	4 (66.7)	2 (33.3)	1 (16.7)	5 (83.3)	4 (66.7)
17	113	112 (99.1)	0	0	3 (2.7)	113 (100.0)	2 (1.8)
18	40	40 (100.0)	0	37 (92.5)	2 (5.0)	40 (100.0)	37 (92.5)
21	39	38 (97.4)	0	0	1 (2.6)	38 (97.4)	0
22	14	0	0	0	0	10 (71.4)	0
24	25	0	0	0	0	20 (80.0)	0
25	17	0	0	0	0	17 (100.0)	0
26	19	0	19 (100.0)	0	0	19 (100.0)	17 (89.5)
30	15	1 (6.7)	0	0	1 (6.7)	9 (60.0)	1 (6.7)
32	5	0	0	0	0	0	0
33	15	3 (20.0)	0	0	0	4 (26.7)	0
34	8	0	0	0	0	8 (100.0)	0
36	8	0	0	0	0	7 (87.5)	0
37	49	41 (83.7)	0	8 (16.3)	0	48 (98.0)	8 (16.3)
38	8	0	0	0	0	0	0
41	62	62 (100.0)	0	5 (8.1)	1 (1.6)	62 (100.0)	5 (8.1)
48	21	21 (100.0)	0	0	0	20 (95.2)	0
49	8	0	0	0	0	0	0
51	12	0	0	0	0	0	0
52	16	6 (37.5)	0	0	0	3 (18.8)	0
54	16	7 (43.8)	0	0	0	7 (43.8)	0
56	25	0	0	0	0	5 (20.0)	0
67	15	0	0	0	0	5 (33.3)	0
68	14	0	0	0	0	0	0

77	29	29 (100.0)	0	0	0	18 (62.1)	0
79	10	10 (100.0)	0	0	0	10 (100.0)	0
90	15	15 (100.0)	0	0	0	15 (100.0)	0
93	6	0	0	0	0	0	0
114	7	0	0	0	0	6 (85.7)	0
125	6	0	0	0	0	4 (66.7)	0
145	8	0	0	0	0	8 (100.0)	0
168	6	6 (100.0)	0	0	0	6 (100.0)	0
205	8	8 (100.0)	0	0	0	8 (100.0)	0
Others[b]	141	33 (23.4)	2 (1.4)	8 (5.7)	3 (2.1)	75 (53.2)	16 (11.3)

[a]GPSC, Global Pneumococcal Sequence Cluster.
[b]Lineages with <5 isolates were not listed individually.

Table 19. Prevalence of antibiotic resistance by pneumococcal lineage in the USA

		Number of isolates (%)					
GPSC[a]	n	Penicillin	Chloramphenicol	Erythromycin-*ermB*	Erythromycin-*mefA*	Cotrimxoazole	Tetracycline
1	84	83 (98.8)	0	80 (95.2)	84 (100.0)	84 (100.0)	83 (98.8)
3	48	0	0	1 (2.1)	24 (50.0)	29 (60.4)	1 (2.1)
4	72	43 (59.7)	0	0	16 (22.2)	25 (34.7)	1 (1.4)
5	12	11 (91.7)	0	2 (16.7)	1 (8.3)	7 (58.3)	2 (16.7)
6	25	15 (60.0)	0	1 (4.0)	8 (32.0)	15 (60.0)	1 (4.0)
7	8	1 (12.5)	0	0	0	2 (25.0)	0
9	15	14 (93.3)	0	12 (80.0)	0	8 (53.3)	15 (100.0)
11	8	0	0	0	2 (25.0)	5(62.5)	0
12	17	0	1 (5.9)	1 (5.9)	0	0	1 (5.9)
15	55	0	0	0	4 (7.3)	0	0
16	17	10 (58.8)	9 (52.9)	5 (29.4)	5 (29.4)	11 (64.7)	10 (58.8)
18	26	17 (65.4)	0	0	24 (92.3)	21 (80.8)	0
19	42	0	0	1 (2.4)	6 (14.3)	0	2 (4.8)
23	11	6 (54.5)	0	1 (9.1)	5 (45.5)	9 (81.8)	4 (36.4)
24	8	1 (12.5)	0	0	1 (12.5)	1 (12.5)	0
27	32	18 (56.3)	0	1 (3.1)	3 (9.4)	0	0
32	22	0	0	0	4 (18.2)	1 (4.5)	0
35	7	0	0	0	0	0	1 (14.3)
36	10	0	0	0	1 (10.0)	1 (10.0)	0
38	17	0	0	0	0	0	0
39	23	0	0	0	3 (13.0)	3 (13.0)	0
50	14	0	0	0	0	1 (7.1)	0
59	15	15 (100.0)	0	0	12 (80.0)	0	0
97	11	11 (100.0)	0	0	11 (100.0)	11 (100.0)	0
119	6	0	0	0	0	0	0
132	8	8 (100.0)	0	0	8 (100.0)	8 (100.0)	8 (100.0)
135	6	0	0	0	0	0	0
Others[b]	55	14 (25.5)	1 (1.8)	2 (3.6)	5 (9.1)	20 (36.4)	8 (14.5)

[a]GPSC, Global Pneumococcal Sequence Cluster.

[b]Lineages with <5 isolates were not listed individually.

Table 20. The top ten and significant increase Global Pneumococcal Sequence Clusters (GPSCs) that caused non-vaccine serotype invasive pneumococcal disease in the PCV13 period.

Rank	Lineage[a]	Major clonal complex/ PMEN	Pooled incidence rate of NVT IPD in the PCV13 period (95% CI)[b]	No. of NVT isolates in the PCV13 period[b]	Increases in NVT-GPSC[c]	Increases in NVT within VT-GPSC[d]	Observed[e]	%Penicillin Resistance	%non-susceptible to ≥2 classes of antibiotics
1	**GPSC3**	CC53/ PMEN33	2.70 (1.34-5.42)	71	SA$^{8\#}$	-	IL^{33F}, GM^{33F}, US^{33F}	6% (4/71)	21% (15/71)
2	GPSC19	CC433	0.89 (0.28-2.82)	21	-	-	IL^{22F}, US^{22F}	0% (0/21)	0% (0/21)
3	**GPSC11**	CC193	0.86 (0.06-12.61)	32	-	IL$^{19A\text{->}15B/C}$	GM^{21}, ML^{21}, SA^{21}, US$^{15B/C/21}$	3% (1/32)	3% (1/32)
4	**GPSC5**	CC172/ PMEN26	0.66 (0.27-1.62)	28	ML$^{35B/D}$	SA$^{23F\text{->}35B/D\#}$	US$^{23A/6C}$	93% (26/28)	86% (24/28)
5	GPSC38	CC393	0.65 (0.11-3.94)	17	-	-	IL^{38}, SA^{38}, US^{38}	0% (0/17)	0% (0/17)
6	GPSC26	CC989	0.58 (0.21-1.58)	35	-		IL^{12F}, GM^{12F}, ML^{12F}, SA^{12F}	0% (0/35)	100% (35/35)
7	GPSC36	CC2068	0.49 (0.20-1.22)	12	-	-	IL^{10A}, SA^{10A}, US^{10A}	0% (0/12)	0% (0/12)
8	GPSC48	CC12127	0.48 (0.12-1.97)	11	-	-	IL$^{15B/C}$, SA$^{15B/C}$	100% (11/11)	64% (7/11)
9	GPSC9	CC63/ PMEN25	0.47 (0.05-4.00)	13	-	-	HK^{15A}, IL^{15A}, SA^{15A}, US^{15A}	100% (13/13)	100% (13/13)
10	GPSC7	CC439	0.42 (0.07-2.64)	10	-	-	IL^{23A}, SA^{23A}, US$^{23A/23B}$	0% (0/10)	0% (0/10)
12	**GPSC59**	CC558/ PMEN24	0.32 (0.06-1.75)	13	US$^{35B/D}$	-	IL$^{35B/D}$, SA$^{35B/D}$	100% (13/13)	77% (10/13)
15	**GPSC55**	CC3524	0.29 (0.00-80.15)	65	IL^{12F}	-	-	100% (65/65)	0% (0/65)
18	**GPSC168**	CC11766	0.22 (0.09-0.50)	5	SA^{15A}	-	-	100% (5/5)	100% (5/5)
19	**GPSC25**	CC8687	0.21 (0.03-1.40)	12	SA$^{15B/C}$	-	-	0% (0/12)	0% (0/12)
29	**GPSC6**	CC156/ PMEN3	0.16 (0.03-0.82)	8	-	US$^{9V\text{->}15B/15C\#}$	HK$^{15B/15C/23A}$, SA^{11A}	25% (2/8)	25% (2/8)
31	**GPSC139**	CC6524	0.16 (0.00-8.87)	9	IL^{10B}	-	-	0% (0/9)	0% (0/9)

36	**GPSC132**	CC172	0.12 (0.01-1.04)	5	US$^{15B/15C\#}$	-	-	100% (5/5)	100% (5/5)

PMEN, Pneumococcal molecular epidemiology clone; CC, Clonal complex; CI, confidence interval; IL, Israel; GM, The Gambia; ML, Malawi; SA, South Africa; US, the United States. Serotypes with a significantly higher invasive disease potential (odds ratio for invasiveness >1, p value <0·05 [A: cite ref 13 here?]) were serotypes 8, 19A, and 12F. Serotypes with a significantly lower invasive disease potential (odds ratio for invasiveness <1, p value <0·05) were 15A, 15B/C, 23B, 21 and 35B.

[a]Significant increases in NVT-GPSCs (>50% NVT in the pre-PCV period) or GPSCs with significant increases in NVT component in VT-GPSCs (≥50% VT in the pre-PCV period) between the pre-PCV and post-PCV periods are bolded. The increases were detected in incidence for Israel, South Africa and the USA while increases in prevalence for Hong Kong, Malawi and The Gambia.

[b]The pooled incidence rates of NVT IPD in the PCV13 period were calculated based on the estimated incidence rate from Israel, South Africa and the USA.

[c]Data are presented as country^{serotype}. Significant increases which only observed between the PCV7 and PCV13 period are marked with #

dData are presented as country $^{\text{predominant serotype in pre-PCV period} \rightarrow \text{predominant serotype in PCV13 period}}$

[e]Lineages observed but without significant increase

Table 21. Differences in prevalence of antibiotic nonsusceptibility within a Global Pneumococcal Sequence Cluster (GPSC) between countries among the top ten GPSCs contributing to NVT IPD in the PCV13 period

	% (no. of isolates)						
Non-susceptibility	Hong Kong	Israel	Malawi	South Africa	The Gambia	USA	P value[a]
GPSC3 (n)	0	14	0	39	2	18	
Penicillin	0	29% (4)	0	0	0	0	0.0039*
Chloramphenicol	0	0	0	0	0	0	ND
Erythromycin	0	14% (2)	0	3% (1)	0	72% (13)	<0.0001*
Cotrimoxazole	0	14% (2)	0	8% (3)	100% (2)	61% (11)	<0.0001*
Tetracycline	0	14% (2)	0	3% (1)	0	5% (1)	0.2413
GPSC5 (n)	0	11	4	22	0	3	
Penicillin	0	100% (11)	100% (4)	95% (21)	0	67% (2)	0.2808
Chloramphenicol	0	0	0	0	0	0	ND
Erythromycin	0	36% (4)	0	5% (1)	0	67% (2)	0.0113*
Cotrimoxazole	0	82% (9)	100% (4)	100% (22)	0	0	0.0002*
Tetracycline	0	0	0	0	0	33% (1)	0.0750
GPSC11 (n)	0	27	1	7	1	2	
Penicillin	0	19% (5)	0	0	100% (1)	0	0.2365
Chloramphenicol	0	0	0	0	0	0	ND
Erythromycin	0	0	0	0	0	0	ND
Cotrimoxazole	0	81% (22)	100% (1)	43% (3)	100% (1)	50% (1)	0.1719
Tetracycline	0	0	0	0	0	0	ND
GPSC9 (n)	1	7	0	2	4	3	
Penicillin	100% (1)	100% (7)	0	100% (2)	100% (4)	100% (3)	1.0000

Chloramphenicol	0	0	0	0	0	0	ND
Erythromycin	100% (1)	100% (7)	0	100% (2)	0	100% (3)	0.0008*
Cotrimoxazole	0	0	0	0	100% (4)	100% (3)	0.0002*
Tetracycline	100% (1)	100% (7)	0	100% (2)	100% (4)	100% (3)	1.0000
GPSC26 (n)	0	3	1	10	21	0	
Penicillin	0	0	0	0	0	0	ND
Chloramphenicol	0	67% (2)	0	100% (10)	90% (19)	0	0.0697
Erythromycin	0	0	0	0	0	0	ND
Cotrimoxazole	0	100% (3)	100% (1)	100% (10)	100% (21)	0	1.0000
Tetracycline	0	100% (3)	100% (1)	90% (9)	90% (19)	0	1.0000
GPSC48 (n)	0	4	0	7	0	0	
Penicillin	0	100% (4)	0	100% (7)	0	0	1.0000
Chloramphenicol	0	0	0	0	0	0	ND
Erythromycin	0	0	0	0	0	0	ND
Cotrimoxazole	0	0	0	100% (7)	0	0	0.0030*
Tetracycline	0	0	0	0	0	0	ND

[a]The prevalence of antibiotic non-susceptibility were compared by Fisher's Exact test. Countries without isolates belong to the tested GPSC are excluded from the comparison. A two-sided p value < 0.05 are considered to be statistically significant and asterisked. ND, statistical test was not performed.

GPSC7, 19, 36 and 38 were not included in this table because they were pan-susceptible to all classes of antibiotics.

Table 22. Changes in antimicrobial nonsusceptibility in GPSC3 and GPSC5 between vaccine periods in the USA and Israel

			Number of isolates (%)			P value		
Country	GPSC	Antibiotic	Pre-PCV period	PCV7 period	PCV13 period	Pre-PCV vs PCV7	PCV7 vs PCV13	Pre-PCV vs PCV13
USA	GPSC3	n	4	26	18	-	-	-
		Penicillin	0	0	0	1.0000	1.0000	1.0000
		Chloramphenicol	0	0	0	1.0000	1.0000	1.0000
		Erythromycin-*mefA*	0	12 (46)	12 (67)	0.1297	0.2268	0.0287*
		Erythromycin-*ermB*	0	0	1 (6)	1.0000	0.4091	1.0000
		Cotrimoxazole	1 (25)	17 (65)	11 (61)	0.2742	1.0000	0.2932
		Tetracycline	0	0	1 (6)	1.0000	0.4091	1.0000
USA	GPSC5	n	0	9	3	-	-	-
		Penicillin	0	9 (100)	2 (67)	1.0000	0.2500	1.0000
		Chloramphenicol	0	0	0	-	-	-
		Erythromycin-*mefA*	0	0	1 (33)	-	1.0000	1.0000
		Erythromycin-*ermB*	0	1 (11)	1 (33)	1.0000	0.4545	1.0000
		Cotrimoxazole	0	7 (78)	0	1.0000	1.0000	-
		Tetracycline	0	1 (11)	1 (33)	1.0000	1.0000	0.4545
Israel	GPSC3	n	11	2	14	-	-	-
		Penicillin	5 (45)	1 (50)	4 (29)	1.0000	1.0000	0.4341
		Chloramphenicol	0	0	0	-	-	-
		Erythromycin-*mefA*	0	0	0	-	-	-
		Erythromycin-*ermB*	0	0	2 (14)	-	1.0000	1.0000
		Cotrimoxazole	1 (9)	0	2 (14)	1.0000	1.0000	1.0000
		Tetracycline	0	0	2 (14)	-	1.0000	1.0000
Israel	GPSC5	n	25	4	11	-	-	-

		Penicillin	25 (100)	4 (100)	11 (100)	1.0000	1.0000	1.0000
		Chloramphenicol	0	0	0	-	-	-
		Erythromycin-*mefA*	2	0	4	1.0000	0.5165	1.0000
		Erythromycin-*ermB*	0	0	0	-	-	-
		Cotrimoxazole	20	3	9	1.0000	1.0000	1.0000
		Tetracycline	0	0	0	-	-	-

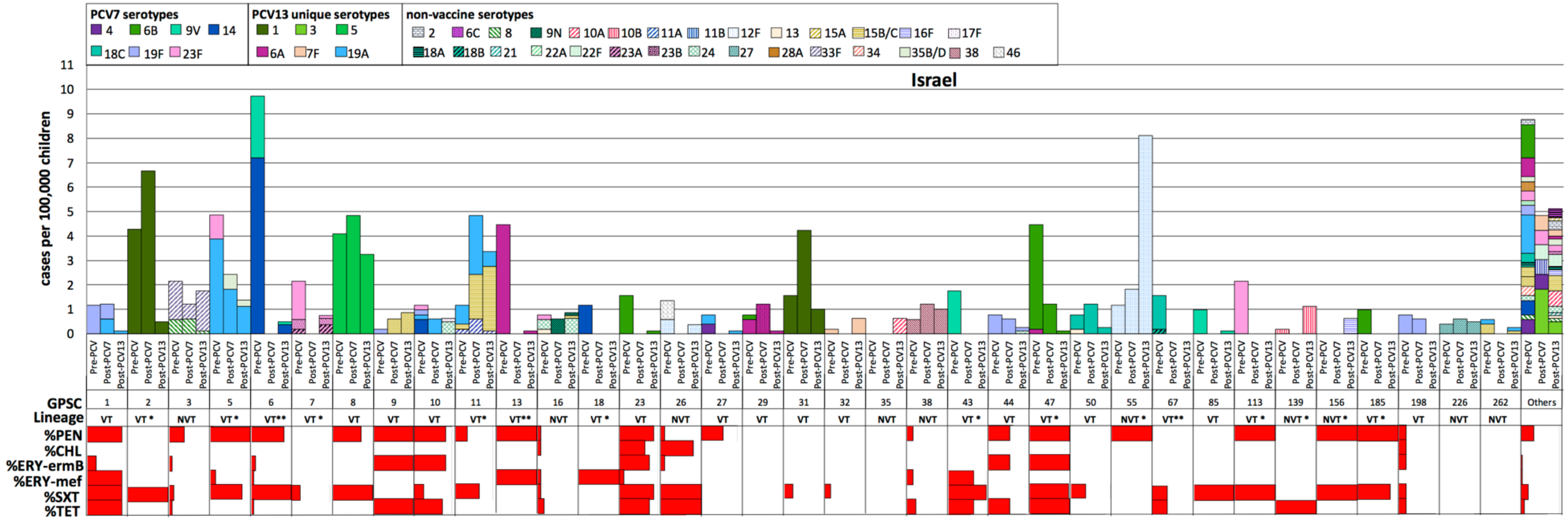

Figure 1. Dynamics of Global Pneumococcal Sequence Clusters (GPSCs) among invasive isolates from children aged <3 years over vaccine periods in Israel. The incidence of invasive pneumococcal disease per 100,000 children aged <3 years is plotted by GPSCs that represented 90% of the Israeli collection, with stratification into three vaccine periods (pre-PCV, post-PCV7, post-PCV13) and coloured by serotypes. Vaccine serotypes are represented by solid fill while NVT by coloured hatched patterns. Underneath the graph, status of a lineage was designated as VT (≥50% VT in the pre-PCV period) or NVT (>50% NVT in the pre-PCV period). Lineages with <5 isolates were not shown. The presence of the first and second asterisk represented a significant change in incidence between the pre-PCV and PCV7 or PCV13 period, respectively. The horizontal bars in red indicate the percentage of antibiotic resistance in each lineage. %PEN, penicillin resistance predicted based on the *pbp1a*, *pbp2x*, *pbp2b* sequences; %CHL, chloramphenicol resistance is predicted by the presence of chloramphenicol acetyltransferase gene, *cat*; %ERY-ermB and %ERY-mefA, macrolide resistance is predicted by the presence of erythromycin resistance methylase gene *ermB* or macrolide efflux pump gene *mefA*; SXT, cotrimoxazole non-susceptibility was determined by the presence of mutation I100L in *folA* and/or any indel within amino acid residue 56-67 in *folP*; TET, tetracycline resistance is predicted by the presence of *tet*M or *tet*(S/M) gene without disruption in the promoter region.

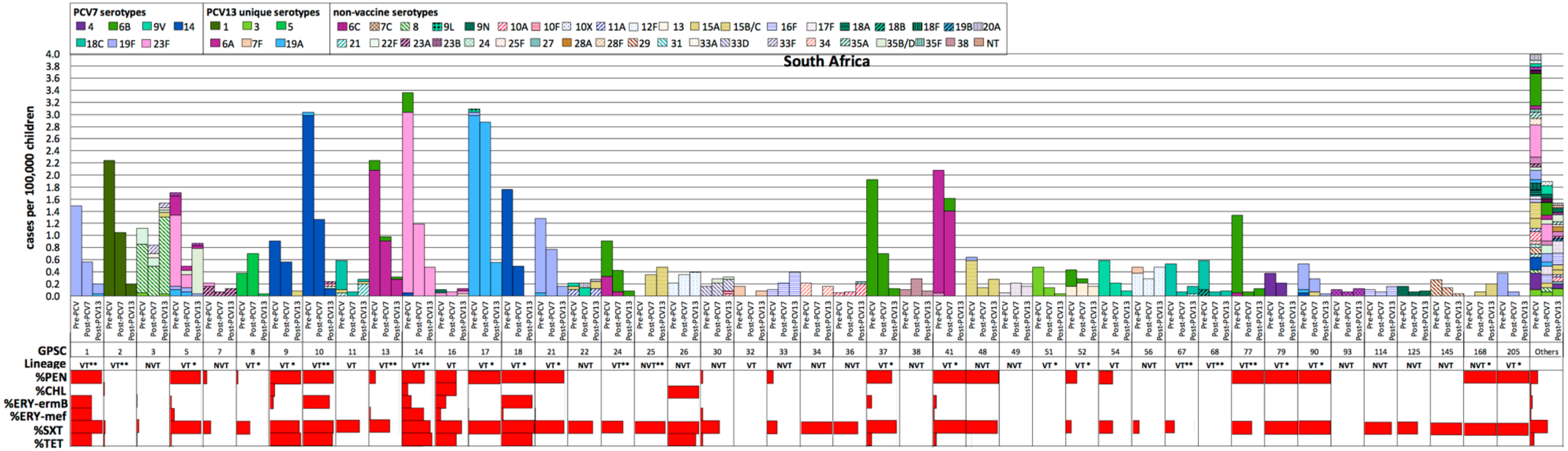

Figure 2. Dynamics of Global Pneumococcal Sequence Clusters (GPSCs) among invasive isolates from children aged <3 years over vaccine periods in South Africa. The incidence of invasive pneumococcal disease per 100,000 children aged <3 years is plotted by GPSCs that represented 90% of the South African collection, with stratification into three vaccine periods (pre-PCV, post-PCV7, post-PCV13) and coloured by serotypes. Vaccine serotypes (VT) are represented by solid fill while non-vaccine serotypes (NVT) by coloured hatched patterns. Underneath the graph, status of a lineage was designated as VT (≥50% VT in the pre-PCV period) or NVT (>50% NVT in the pre-PCV period). Lineages with <5 isolates were not shown. The presence of the first and second asterisk represented a significant change in incidence between the pre-PCV and PCV7 or PCV13 period, respectively. The horizontal bars in red indicate the percentage of antibiotic resistance in each lineage. %PEN, penicillin resistance predicted based on the *pbp1a*, *pbp2x*, *pbp2b* sequences; %CHL, chloramphenicol resistance is predicted by the presence of chloramphenicol acetyltransferase gene, *cat*; %ERY-ermB and %ERY-mefA, macrolide resistance is predicted by the presence of erythromycin resistance methylase gene *ermB* or macrolide efflux pump gene *mefA*; SXT, cotrimoxazole non-susceptibility was determined by the presence of mutation I100L in *folA* and/or any indel within amino acid residue 56-67 in *folP*; TET, tetracycline resistance is predicted by the presence of *tet*M or *tet*(S/M) gene without disruption in the promoter region.

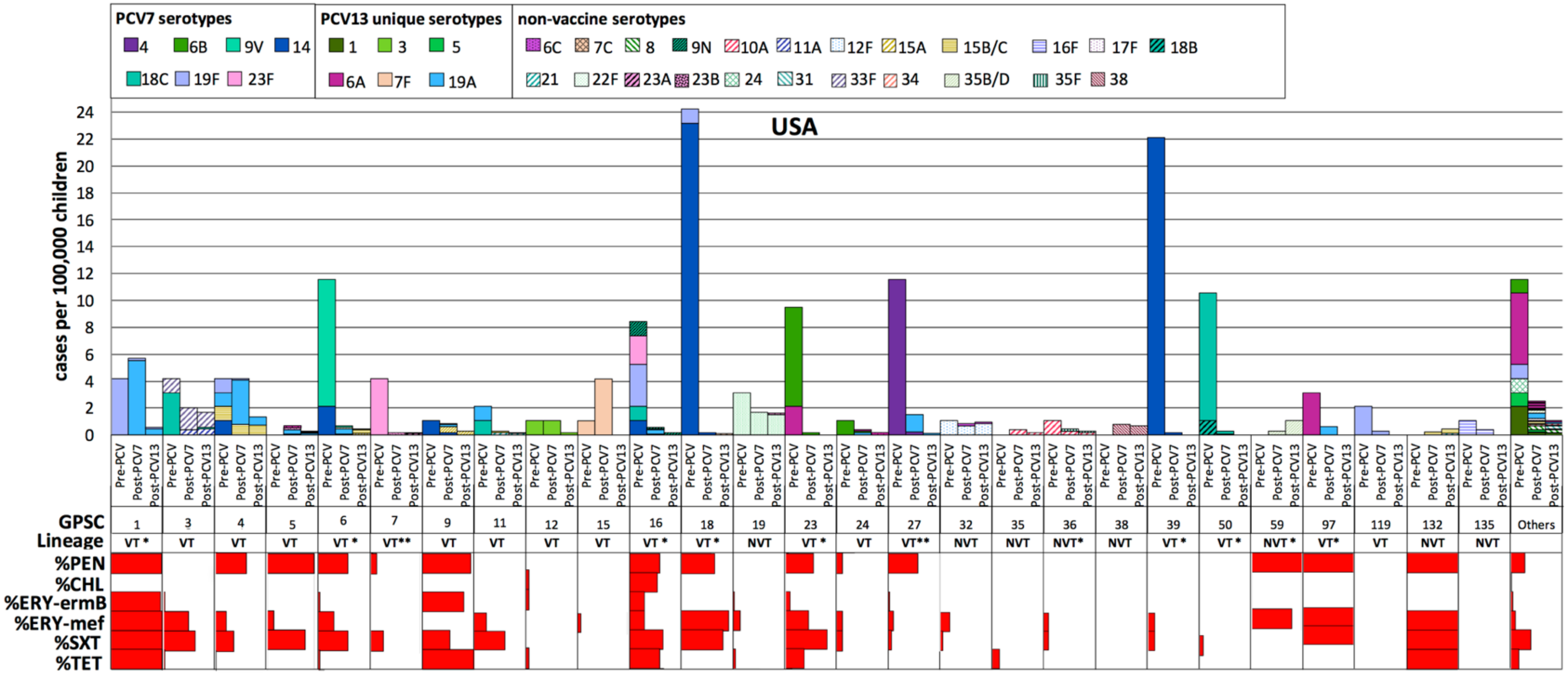

Figure 3. Dynamics of Global Pneumococcal Sequence Clusters (GPSCs) among invasive isolates from children aged <3 years over vaccine periods in the USA. The incidence of invasive pneumococcal disease per 100,000 children aged <3 years is plotted by GPSCs that represented 90% of the American collection, with stratification into three vaccine periods (pre-PCV, post-PCV7, post-PCV13) and coloured by serotypes. Vaccine serotypes are represented by solid fill while NVT by coloured hatched patterns. Underneath the graph, status of a lineage was designated as VT (≥50% VT in the pre-PCV period) or NVT (>50% NVT in the pre-PCV period). Lineages with <5 isolates were not shown. The presence of the first and second asterisk represented a significant change in the average incidence between the pre-PCV and PCV7 or PCV13 period, respectively. The horizontal bars in red indicate the percentage of antibiotic resistance in each lineage. %PEN, penicillin resistance predicted based on the *pbp1a*, *pbp2x*, *pbp2b* sequences; %CHL, chloramphenicol resistance is predicted by the presence of chloramphenicol acetyltransferase gene, *cat*; %ERY-ermB and %ERY-mefA, macrolide resistance is predicted by the presence of erythromycin resistance methylase gene *ermB* or macrolide efflux pump gene *mefA*; SXT, cotrimoxazole non-susceptibility was determined by the presence of mutation I100L in *folA* and/or any indel within amino acid residue 56-67 in *folP*; TET, tetracycline resistance is predicted by the presence of *tet*M or *tet*(S/M) gene without disruption in the promoter region.

References:

1. Gladstone RA, Lo SW, Lees JA, Croucher NJ, van Tonder AJ, Corander J, et al. International genomic definition of pneumococcal lineages, to contextualise disease, antibiotic resistance and vaccine impact. EBioMedicine. 2019.

2. Cameron AC, Trivedi PK. Microeconometrics using Stata. College Station, Tex.: Stata Press; 2009.

3. Berger S, Graham N, Zeileis A. Various Versatile Variances: An Object-Oriented Implementation of Clustered Covariances in R. Faculty of Economics and Statistics, University of Innsbruck; 2017.

4. Miller E, Andrews NJ, Waight PA, Slack MP, George RC. Herd immunity and serotype replacement 4 years after seven-valent pneumococcal conjugate vaccination in England and Wales: an observational cohort study. Lancet Infect Dis. 2011;11(10):760-8.

5. Mark Stevenson TN, Cord Heuer, Jonathon Marshall, Javier Sanchez, Ron Thornton, Jeno Reiczigel, Jim Robison-Cox, Paola Sebastiani, Peter Solymos, Kazuki Yoshida, Geoff Jones, Sarah Pirikahu, Simon Firestone, Ryan Kyle, Johann Popp, and Mathew Jay. epiR: Tools for the Analysis of Epidemiological Data. R Foundation for Statistical Computing; 2017.
